# Effects of galloflavin and ellagic acid on sirtuin 6 and its anti-tumorigenic activities

**DOI:** 10.1016/j.biopha.2020.110701

**Published:** 2020-09-06

**Authors:** Minna Rahnasto-Rilla, Joni Järvenpää, Marjo Huovinen, Anna-Mari Schroderus, Emmi-Leena Ihantola, Jenni Küblbeck, Mohammed Khadeer, Ruin Moaddel, Maija Lahtela-Kakkonen

**Affiliations:** a School of Pharmacy, University of Eastern Finland, 70210, Kuopio, Finland; b Department of Clinical Microbiology, Institute of Clinical Medicine, University of Eastern Finland, Kuopio, Finland; c Biomedical Research Center, National Institute on Aging, National Institutes of Health, Baltimore, Maryland, 21224, USA

**Keywords:** Berries, Cancer, Polyphenol, Sirtuin, Tannin

## Abstract

Sirtuin 6 (SIRT6), a member of sirtuin family (SIRT1–7), regulates distinct cellular functions; genome stability, DNA repair, and inflammation related diseases. Recently, we demonstrated that anthocyanidins in berries induce the catalytic activity of SIRT6. In this study, we explored the effects of Galloflavin and Ellagic acid, the most common polyphenols in berries, on SIRT6.

SIRT6 deacetylation was investigated using HPLC and immunoblotting assays. The expression levels of SIRT6, glycolytic proteins and cellular metabolism were studied on human colon adenocarcinoma cells (Caco2). Molecular docking studies were carried out to study possible interactions of the compounds with sirtuins.

Ellagic acid increased the deacetylase activity of SIRT6 by up to 50-fold; it showed moderate inhibition of SIRT1–3. Galloflavin and Ellagic acid showed anti-proliferative effects on Caco2. The compounds also upregulated SIRT6 expression whereas key proteins in glycolysis were downregulated. Galloflavin decreased glucose transporter 1 (GLUT1) expression, and Ellagic acid affected the expression of protein dehydrogenase kinase 1 (PDK1). Interestingly, both compounds caused reduction in glucose uptake and lactate production. Both Galloflavin and Ellagic acid were able to form hydrogen bonds with Asp188 and Gly6 in SIRT6.

In this study, we showed that Galloflavin and Ellagic acid increased SIRT6 activity and decreased the expression of SIRT6 associated proteins involved in cancer development. Taken together, Galloflavin and Ellagic acid targeting SIRT6 activity may provide a new insight in the development of anti-cancer therapy.

## 1. Introduction

Sirtuin 6 (SIRT6), a nicotinamide adenine dinucleotide (NAD^+^)-dependent deacetylase, is a powerful epigenetic regulator, which affects various cellular processes; glucose homeostasis, telomere maintenance, and DNA repair [1,2]. SIRT6 has been shown to possess deacetylation [3,4], mono-ADP-ribosyltransferase [5] and deacylation activities [6]. SIRT6 deacetylation activity has been associated with glucose homeostasis and it functions as a histone 3 lysine 9 (H3K9) deacetylase affecting gluconeogenesis, increasing mitochondrial respiration, and inhibiting glycolysis [7]. Glycolysis is controlled by SIRT6 via hypoxia-inducible factor 1α (HIF1α), which is a critical regulator of nutrient and oxygen stress responses [8]. The deficiency of SIRT6 demonstrates the stimulation of HIF1α activity, and thus increases the expression of glycolysis-related genes, such as glucose transporters (GLUTs), lactate dehydrogenase (LDH), and pyruvate dehydrogenase kinases (PDKs) [8]. SIRT6 indirectly inhibits gluconeogenesis by inactivating a key transcriptional regulator of gluconeogenesis, peroxisome proliferator-activated receptor γ coactivator 1-alpha (PGC-1α) through general control non-repressed 5 (GCN5) activity [9].

Most cancer cells exhibit an altered metabolism with increased glycolysis, also known as the Warburg effect, which is important for supporting rapid tumor growth. SIRT6 activation inhibits this glycolytic shift [7], and thus SIRT6 deficiency increases cell survival, growth and proliferation in many cancers [1,2]. SIRT6 deficiency occurs in several human cancers, particularly in pancreatic and colorectal cancer cells [2, 7]. Overall, SIRT6 can act as a tumor suppressor, and an increase in SIRT6 activity or protein levels contributes to a tumor suppression, indicating a promising strategy for cancer prevention.

**Fig. 1.**
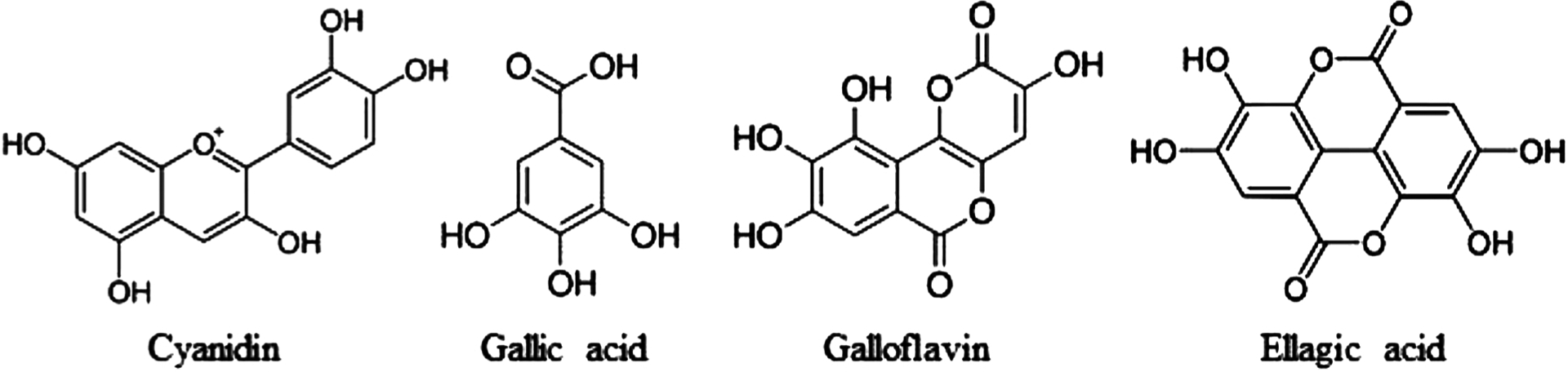
The chemical structures of Cyanidin, Gallic acid, Galloflavin and Ellagic acid.

Various polyphenols have known epigenomic modifying activities. Our recent findings demonstrate that a group of the naturally occurring pigments in berries, also known as anthocyanidins, induced the catalytic activity of SIRT6 *in vitro* [10]. Cyanidin (Fig. 1), belonging to anthocyanins, increased SIRT6 protein expression in human colon adenocarcinoma (Caco2) cells. Among the many polyphenols found in berries, the most abundant of them are hydrolysable tannins. They can be further classified as gallotannins that are hydrolyzed into Gallic acid and ellagitannins that are hydrolyzed into Ellagic acid (Fig. 1) [11]. Ellagitannins occur especially in berries growing in Artic regions, such as cloudberry (*Rubus chamaemorus*) and arctic bramble (*Rubus arcticus*) [12]. Bioactive compounds in berries appear to act through critical cell signaling pathways that influence many physiological processes. For example, cloudberry contains a number of polyphenolic compounds that are strong antioxidants and potential anticarcinogenic agents [13]. Hydrolysable tannins and their hydrolytic products exhibit also anti-cancer activities through mechanisms not fully understood.

In this study, we investigated the effects of Gallic acid, Galloflavin and Ellagic acid (Fig. 1) on SIRT6 deacetylation and expression *in vitro*. The expression levels of glycolytic proteins and cellular metabolism were studied in Caco2 cells. Ellagic acid induced SIRT6 activity, decreased the PDK1 levels, reduced glucose uptake and lactate production. The interactions between compounds and various sirtuins were explored by molecular docking.

## 2. Experimental section

### 2.1. Material

Acetylated histone (H3K9Ac) peptide was from AnaSpec (USA). Fetal bovine serum (FBS), Novex 10–20 % gradient gels, anti-GLUT1-IgG (PA516793), anti- PDK1-IgG (PA528342), anti-SIRT6-IgG (PA517215), and anti-rabbit-IgG (mouse) Horseradish peroxidase (HRP)-conjugated secondary antibody (G21234) were from Life Technologies (UK). Ellagic acid, formic acid, Gallic acid, Galloflavin, NAD^+^, propidium iodide (PI) and anti-α-Tubulin-IgG (T5168) were from Sigma-Aldrich (USA). Anti-mouse-IgG (rabbit) HRP-conjugated secondary antibody (ab97046) was from Abcam (UK). Dulbecco modified Eagle medium (DMEM) and non-essential amino acids were from Lonza (Belgium). Anti-acetyl H3K9-IgG, purified chicken core histones (13–107) and digitonin (Calbiochem: 300,410) were from Millipore (USA). Anti-histone H3-IgG (9715) was from Cell Signaling Technology (Netherlands). Laemmli SDS sample buffer (J61337) was from Alfa Aesar, Thermo Fisher Scientific (German) and RNase A (EN0531) was from Thermo Fisher Scientific (German). Enhanced chemiluminescence (ECL) prime western blotting detection reagents were from Amersham BioSciences (UK). Penicillin/Streptomycin was from EuroClone (Italy). l-Glutamine was from Bio-west (France).

The human SIRT6 expression vector hSIRT6-pGEX-6P3 was kindly provided by Prof. Katrin Chua (Stanford, USA). Recombinant glutathione-S-transferase (GST)-tagged SIRT6 was produced by fermentation in *E*. *coli* BL21(DE3)-pRARE at +16 °C with 0.1 mM IPTG for 20 -h and the soluble overexpressed protein was purified on glutathione agarose (Sigma, USA).

Radioimmunoprecipitation assay (RIPA) lysis buffer was prepared in 50 mM Tris–HCl buffer (pH = 8.0) consisting of 150 mM NaCl, 1% NP-40, 0.5 % Na-deoxycholate, 5 mM EDTA, 0.1 % SDS.

### 2.2. In vitro assays

#### 2.2.1. SIRT6 HPLC

The assay was performed as previously reported [14,15]. Briefly, Gallic acid and Galloflavin in DMSO or Ellagic acid in Tris/NaOH and DMSO/Tris/NaOH (control) were incubated for 30 min with GST-SIRT6 (3 μg/well), H3K9Ac (1–225 μM) and 500 μM NAD^+^ in Tris–HCl Buffer (25 mM, pH 8.0) at +37 °C. Control samples for compounds without NAD^+^ or SIRT6 were carried out. The deacetylation reaction was terminated by adding 6 μL of cold 10 % formic acid and centrifuged for 15 min. The samples were analyzed by reversed-phase HPLC. The formation of deacetylated product (H3K9) and substrate (H3K9Ac) peaks was monitored and subsequently quantified by measuring area under the curve. Experiments were repeated in triplicate, Michaelis-Menten analysis, K_m_ and EC_50_values were calculated using Graph Pad Prism Software version 6 (California, USA).

#### 2.2.2. SIRT6 immunoblotting assay

The assay was carried out as previously described with slight modifications [16]. Briefly, the compounds and DMSO/Tris/NaOH control were incubated for 30 min in the presence of 3 μg of purified recombinant GST-SIRT6, 1.25 μg purified chicken core histones, and 500 μM NAD^+^ in 25 mM Tris–HCl, pH 8.0 at +37 °C. The reaction was stopped with Laemmli (sample buffer) and separated by SDS-PAGE using 10–20 % gradient gels and transferred onto polyvinylidene difluoride (PVDF) membranes. H3K9 acetylation was detected with rabbit anti-acetyl H3K9 antibody followed by anti-rabbit HRP-conjugated secondary antibody. Membranes were stripped and re-probed with rabbit anti-H3 antibody. Chemiluminescent signal detection and image acquisition were carried out using ECL prime western blotting detection reagents. The acetylation status was evaluated by determining the remaining levels of histone H3 acetylated on lysine 9 and normalized to total H3 histone. The results are presented as a fold change compared to the loading control value (mean ± SEM). Statistical significance of treated groups to DMSO control groups were analyzed with one-way ANOVA followed Bonferroni and Dunnett post hoc test.

#### 2.2.3. SIRT1–3 fluor de lys assays

Experiments were performed to determine the activity of Galloflavin and Ellagic acid towards other sirtuins, as previously described [17]. Assays were based on the method described in the BioMol product sheet (Enzo Life Sciences, USA). BioMol KI177 substrate for SIRT1 and KI179 substrate for SIRT2 and SIRT3 were used. Briefly, the reaction was started by incubating the enzyme (SIRT1–3) with the reaction mixture containing acetylated peptide substrate (0.7 Km: 58 μM for SIRT1, 198 μM for SIRT2, and 32 μM for SIRT3), NAD^+^ (0.9 Km: 558 μM for SIRT1, 547 μM for SIRT2, and 2 mM for SIRT3). Incubation was done at 37 °C for 1-h. The developer and nicotinamide (2 mM in histone deacetylase (HDAC) assay buffer giving a total volume of 50 μL) were added and the incubation was continued for 45 min at 37 °C. The fluorescence was measured using EnVision 2104 Multilabel Reader (PerkinElmer, Waltham, MA, USA) with excitation and emission wavelengths of 370 nm and 460 nm, respectively.

### 2.3. Molecular modeling

Modeling studies were performed with Schrödinger Maestro version 11.8 (Schrödinger Release 2018–4, Maestro). Compounds were generated with Maestro and were prepared with Ligand Preparation tool using default settings with OPLS3e force field, possible ionization states were generated with Epic v. 4.6 at pH 7.4 ± 2.0 and a maximum a 10 stereoisomers per ligand were created. Protein structures applied for docking were SIRT1 (PDB:4I5I) [18], SIRT2 (PDB:4RMG) [19], SIRT3 (PDB:4BV3) [20], and SIRT6 (PDB:3ZG6) [6]. Protein structures were prepared with Maestro’s Protein Preparation Wizard -tool. Standard settings were used with all proteins at pH 7.4, all waters were removed, missing sidechains were added using Prime and the protein was refined using OPLS3e force field [21].

Glide was applied for molecular docking studies. Binding sites in each SIRTs were outlined by identifying the region or coordinates of the co-crystallized ligands using grid generators’ (Glide version 8.1) standard settings. In case of SIRT6 the binding site was set based on the myristoyl-moiety of co-crystallized ligand [6] which we have applied previously [10]. SP setting (standard precission) was used in docking.

### 2.4. Cell culture assays

#### 2.4.1. Cell culture and treatments

Caco2 cells (passage 30–40) were cultured in DMEM with 10 % FBS, 1 % nonessential amino acids, 2 mM l-glutamine, 100 units/mL of penicillin, and 100 μg/mL of streptomycin at +37 °C (5 % CO_2_ + air) for 14 days before the treatments. Cells for viability experiments were exposed on 48-well plates (6 × 10^4^ cells/well), with four replicates per concentration. For the immunoblotting, cells were seeded on 24-well plates (1 × 10^5^ cells/well) and for the cell cycle analysis on 6-well plates (7 × 10^5^ cells/well). All the experiments at different time points and concentrations were repeated independently at least three times. After 24-h, cells were treated with 0.5 % DMSO (control) or various concentrations of compound (Galloflavin/Ellagic acid) for 6–24 h. In all experiments, statistical significance of treated groups to DMSO control groups were analyzed with one-way ANOVA followed Bonferroni and Dunnett post hoc test, which all done by using Graph Pad Prism Software version 6 (California, USA).

#### 2.4.2. Cell viability and number

Cell viability measurement was carried out as described previously [22]. This method is based on the fluorescence of PI that can only enter cells and nuclei with damaged membranes, while the membranes of viable cells are impermeable to PI. Briefly, at the end of the exposure to Galloflavin or Ellagic acid, PI was added to each well, incubated for 20 min. In order to carry out cell number, the cells were further treated with digitonin (160 μM), that known to damage the cell wall and the nuclear membrane, making them permeable to PI and incubated for 20 min. Fluorescence was measured both before (effect on viability of compounds) and after (total cell number) the digitonin addition at the excitation wavelength of 531 nm and the emission wavelength of 615 nm using Victor (Wallac, 1420 Multilabel Counter). Background fluorescence values (blank 1 and blank 2) were obtained from cell-free wells after PI and digitonin treatment, respectively. Percent cell viability was calculated using the following equation: 100 – [(F – blank 1)/ (F_MAX_–blank 2)] × 100. F = fluorescence after PI and F_MAX_= fluorescence after digitonin addition. The relative cell numbers were expressed as percentages from control values [(100 × F_MAX_ of exposed / F_MAX_ of the corresponding control)].

#### 2.4.3. Cell cycle analysis by flow cytometry

Cells were collected after 24-h treatment, fixed in ice-cold ethanol/PBS (70:30) overnight at −20 °C. Cells were resuspended in PBS containing 0.15 mg/mL RNase and incubated at 50 °C for 1-h. The DNA content was analyzed by staining the cells with PI (20 μg/mL) and the distribution of the cells in different cell cycle phases was measured with a Novocyte Quanteon flow cytometer (ACEA Biosciences). Data are presented as mean values of three independent experiments.

#### 2.4.4. Immunoblotting

After treatment cells were washed twice with ice-cold PBS. RIPA buffer was added to the cells and incubated for 30 min. Cell suspensions were collected and centrifuged (13,000 rpm, 20 min, +4 °C). Supernatant containing the proteins were aliquoted and stored at −80 °C. Sample protein concentrations were measured with Bradford Assay (Bio-Rad DC^™^ Protein Assay) (Sigma-Aldrich, USA).

Immunoblotting was performed according to standard protocols from three independent experiments. Briefly, protein samples were separated by SDS-PAGE using 10–20 % gradient gels and transferred onto PVDF membranes. Membranes were blocked in 3% non-fat dry milk and further incubated with primary rabbit anti- antibodies overnight at +4 °C. HRP-conjugated secondary antibodies (goat anti-rabbit, goat anti-mouse) were incubated for 1-h at room temperature and proteins were detected using ELC prime western blotting system. Densitometric analysis of protein bands were carried out using ImageJ 1.32 software and the data were normalized by H3 or α-tubulin (loading controls). The results are presented as a fold change compared to the loading control value (mean ± SEM).

#### 2.4.5. Glucose uptake

Glucose uptake was evaluated by exposing the cells to a fluorescence-labeled deoxyglucose analog (2-[N-(7-nitrobenz-2-oxa-1,3-diazol-4-yl) amino]-2-deoxy-d-glucose) (2-NBDG) that can be used as a probe for the detection of glucose taken up by cultured cells [23]. Caco2 cells (2 × 10^4^ cells/well) were seeded on 96-well plates for 24-h. Cells were then glucose starved for 24-h in the presence of Apigenin as a control (data not show), Galloflavin or Ellagic acid followed by incubation with 100 μM of 2-NBDG for 120 min. Cells were harvested and fluorescence intensity was measured using excitation/emission maxima of 485/530 nm (EnVision 2104 Multilabel Reader (PerkinElmer, Waltham, MA, USA).

#### 2.4.6. Lactate production

Lactate concentrations in the culture medium were measured using lactate colorimetric assay kit according to instructions of the manufacturer (Sigma-Aldrich, USA). In the assay lactate production was determined using lactate dehydrogenase to generate a product which interacts with a probe to produce a color that was measured with EnVision 2104 Multilabel Reader (PerkinElmer, Waltham, MA, USA) (λ_max_ = 450 nm).

## 3. Results

### 3.1. Galloflavin and Ellagic acid stimulate SIRT6 deacetylation

**Fig. 2.**
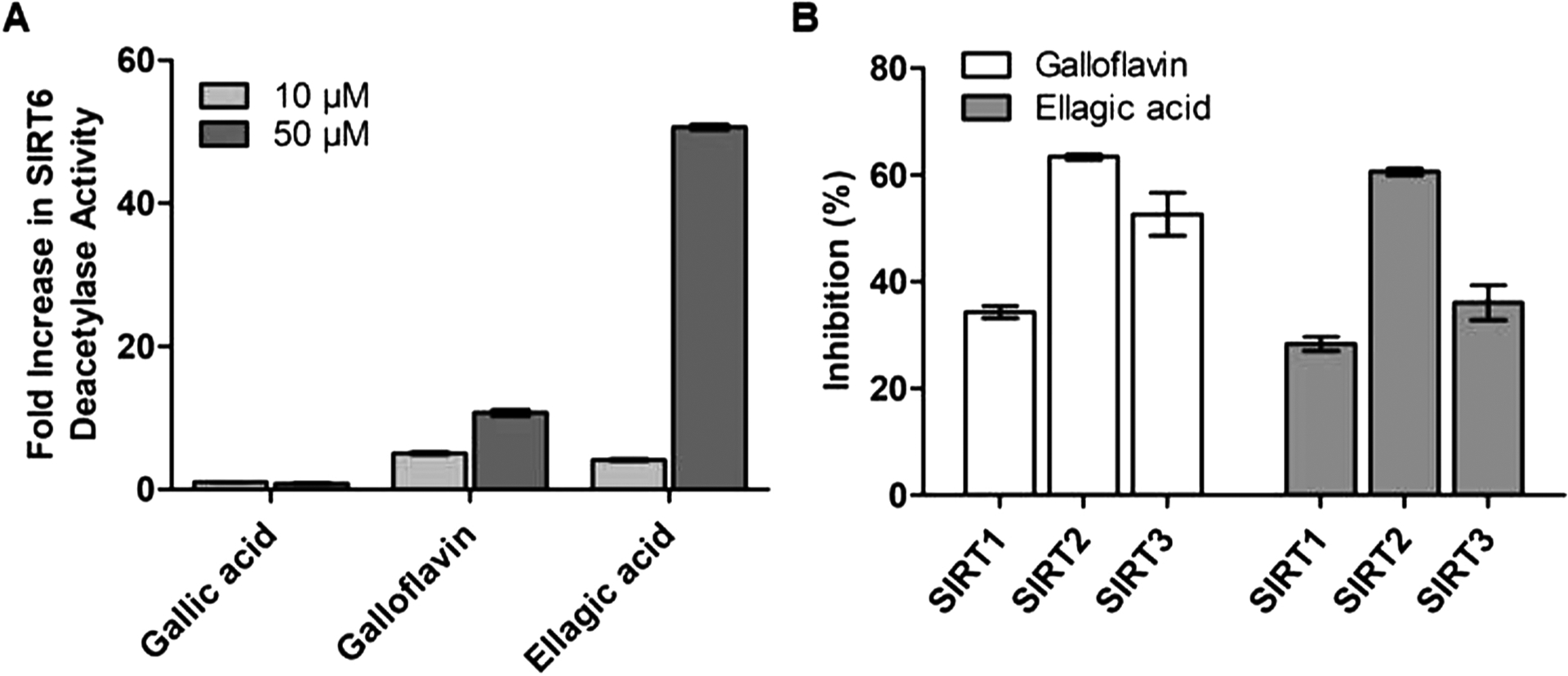
Galloflavin and Ellagic acid stimulate SIRT6 and inhibit SIRT2 deacetylation activities *in vitro*. (A) SIRT6 deacetylation activity in the presence of Gallic acid, Galloflavin and Ellagic acid at 10 μM and 50 μM concentrations. (B) SIRT1–3 deacetylation activity in the presence of Galloflavin and Ellagic acid at 100 μM. The results are presented as a fold change compared to the DMSO control value (mean ± SD; n = 3).

In the present study the experiments were carried out using the concentration range of 0 μM to 100 μM based on literature [24,25]. Ellagic acid showed 50-fold SIRT6 activation whereas Galloflavin produced only approximately 10-fold activation at 50 μM concentration using HPLC based SIRT6 deacetylation assay (Fig. 2A). At lower concentrations, both compounds provided a moderate increase in SIRT6 activation. We also investigated the effects of Galloflavin and Ellagic acid on the deacetylation activity of other SIRTs (Fig. 2B). Surprisingly, opposite to the effect on SIRT6, Galloflavin and Ellagic acid displayed moderate inhibition against SIRT1 and SIRT3, but approximately up to 60 % inhibition against SIRT2 at 100 μM concentration.

**Fig. 3.**
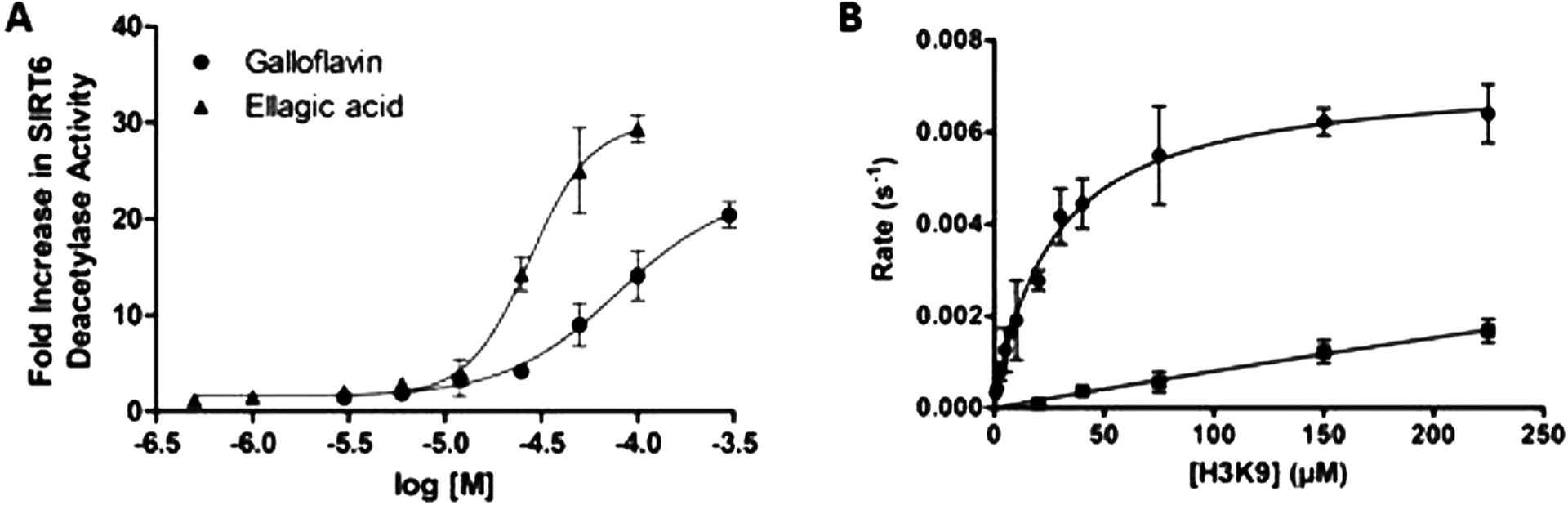
Galloflavin and Ellagic acid dose-dependently induce SIRT6 activity. (A) Dose response effect on SIRT6 deacetylation activity by Galloflavin and Ellagic acid by HPLC assay. (B) Ellagic acid induced a decrease in K_m_ value of acetylated H3K9Ac substrate. Michaelis-Menten analysis of H3K9Ac (0–225 μM) with (filled square) and without (filled circles) Ellagic acid at 25 μM concentrations. The data are presented as mean ± SEM (n = 3).

EC_50_ values for SIRT6 activation were 79 μM and 25 μM for Galloflavin and Ellagic acid (Fig. 3A), respectively. SIRT6 deacetylation activity was also determined for both compounds by immunoblotting analysis using the core histones as a substrate (Fig. S1). H3K9Ac levels were normalized relative to H3, and quantification was presented as fold change with respect to the control. The results showed that both compounds stimulated SIRT6 significantly in a dose dependent manner.

A steady-state kinetic analysis was performed for Ellagic acid in order to obtain kinetic parameters. Ellagic acid demonstrated a dramatic decrease in the Michaelis Menten constant to yield a K_m_ value of 25 μM, and also enhanced catalytic efficiency (K_cat_/K_m_) by 30-fold (Fig. 3B). The experiment was performed by increasing the concentration of H3K9Ac peptide at saturating levels of NAD^+^ in the presence or absence of Ellagic acid 25 μM.

### 3.2. Molecular modeling studies in sirtuins

All sirtuins have the catalytic core region of approximately 275 amino acids [26]. This catalytic core includes Rossmann-fold domain for NAD^+^ binding and zinc-binding domain. There is a large hydrophobic pocket between these domains that can accommodate various compounds capable of modulating deacetylation activity of sirtuins. Molecular docking was applied to study the interactions of Galloflavin, Ellagic acid, and Gallic acid in the hydrophobic pocket of various SIRTs.

**Fig. 4.**
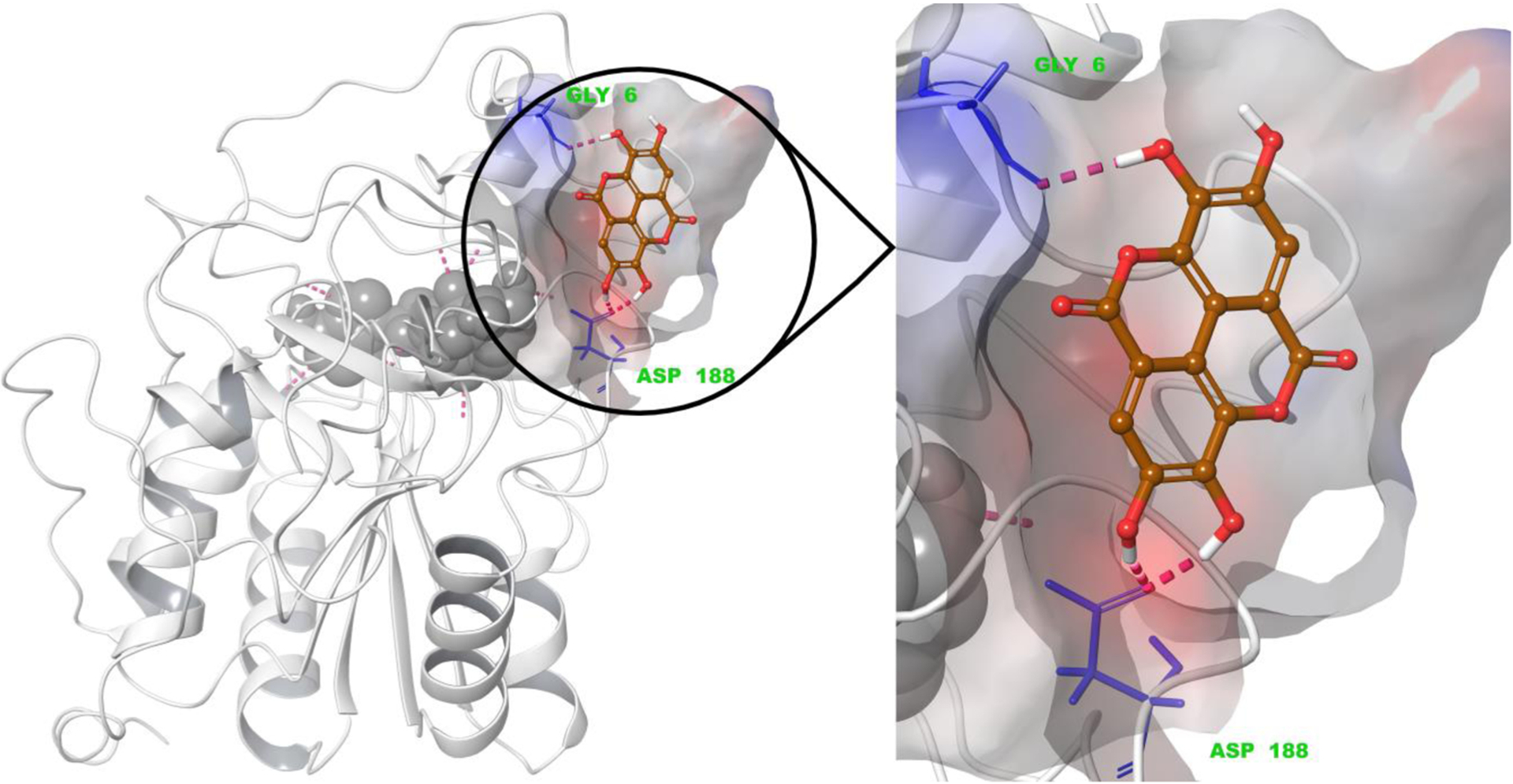
The docking pose of Ellagic acid at SIRT6′s putative activator site. The purple dashes indicate hydrogen bonding between amino acids (Gly6, Asp188) and Ellagic acid. The dark grey molecule is ADP-ribose (Adenosine diphosphate ribose) part of cofactor NAD^+^. The surface is colored by the electrostatic potential with blue showing the highest potentials and red the lowest.

Galloflavin showed a docking pose in the same region of the hydrophobic pocket in SIRT6 as known activators [6], like Cyanidin [10]. Galloflavin formed two hydrogen bonds with Asp188 and one with Gly6 with SIRT6 (Fig. S2). Ellagic acid occupied the same region as Galloflavin and had the same interactions (Fig. 4, Fig. S3). Gallic acid showed two hydrogen bonded interactions with Asp188 and one with Asp185, although as a smaller compound it can occupy various positions in the pocket (Fig. S4).

In SIRT1–3 the compounds shared a docking pose in same region as the co-crystallized ligands [18–20]. Galloflavin displayed three hydrogen bonds and four hydrophobic interactions with SIRT1 (Fig. S5). In case of SIRT2 it showed one hydrogen bond and one hydrophobic interaction (Fig. S6), whereas it formed two hydrogen bonds and had two hydrophobic interactions in SIRT3 (Fig. S7). Ellagic acid had several interactions with SIRT1: two hydrogen bonds and four hydrophobic interactions (Fig. S8). Ellagic acid showed two hydrophobic interactions in SIRT2 (Fig. S9), but with SIRT3 it formed more interactions: three hydrogen bonds and two hydrophobic interactions (Fig. S10). Gallic acid had only one hydrogen bond in each of SIRT1–3, but it had additional hydrophobic interactions with SIRT1 and SIRT3 (Figs. S11, S12, S13).

### 3.3. Galloflavin and Ellagic acid demonstrated inhibition of cell proliferation but an increase in SIRT6 expression in Caco2 cells

**Fig. 5.**
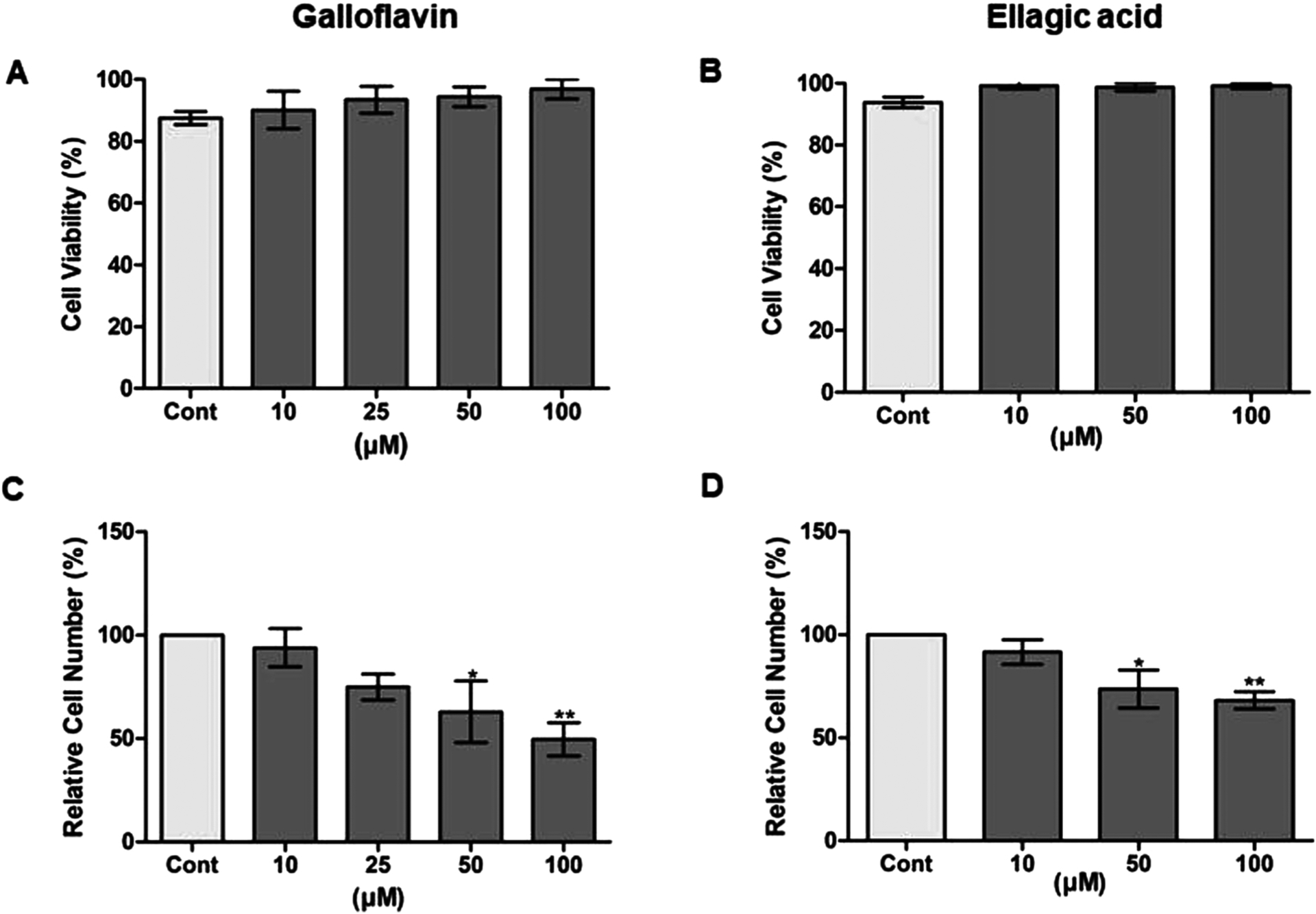
Galloflavin and Ellagic acid did not show effect on cell viabilitybut decreased relative cell numberafter 24-h treatments. (A, B) Cell viability is expressed as percent of viable cells from total number of cells, and(C, D) relative cell number as percent of treated cells (gray bars) from DMSO control (light gray bars). The values are means ±SEM from three individual experiments (n = 3) each mean of four replicates on the same 48-well plate. Statistical significance of treated groups to DMSO control groups were analyzed with one-way ANOVA followed Bonferroni and Dunnett post hoc test (*p values < 0.05 vs. control, **p values < 0.01 vs. control).

According to the cell viability assay, neither Galloflavin nor Ellagic acid affected the cell viability of Caco2 cells at any concentration (Fig. 5A and B). However, both compounds decreased significantly and dose-dependently the relative cell number of Caco2 cells indicating the effect of cell proliferation (Fig. 5C and D).

The effect of Ellagic acid for proliferation was further examined by analyzing the cells in different cell cycle phases (Fig. S14). Control cells were distributed as follows: 49 % in G_0_/G_1_ phase, 28 % in S phase and 18 % in G_2_/M phase. Following treatment with Ellagic acid there was a slight but not significant decrease of cells in G_0_/G_1_ phase and increase of cells in phase S after treatment at the highest concentrations. However, the percentage of cells in G_2_/M phase remained unaltered between the control and the treated cells.

**Fig. 6.**
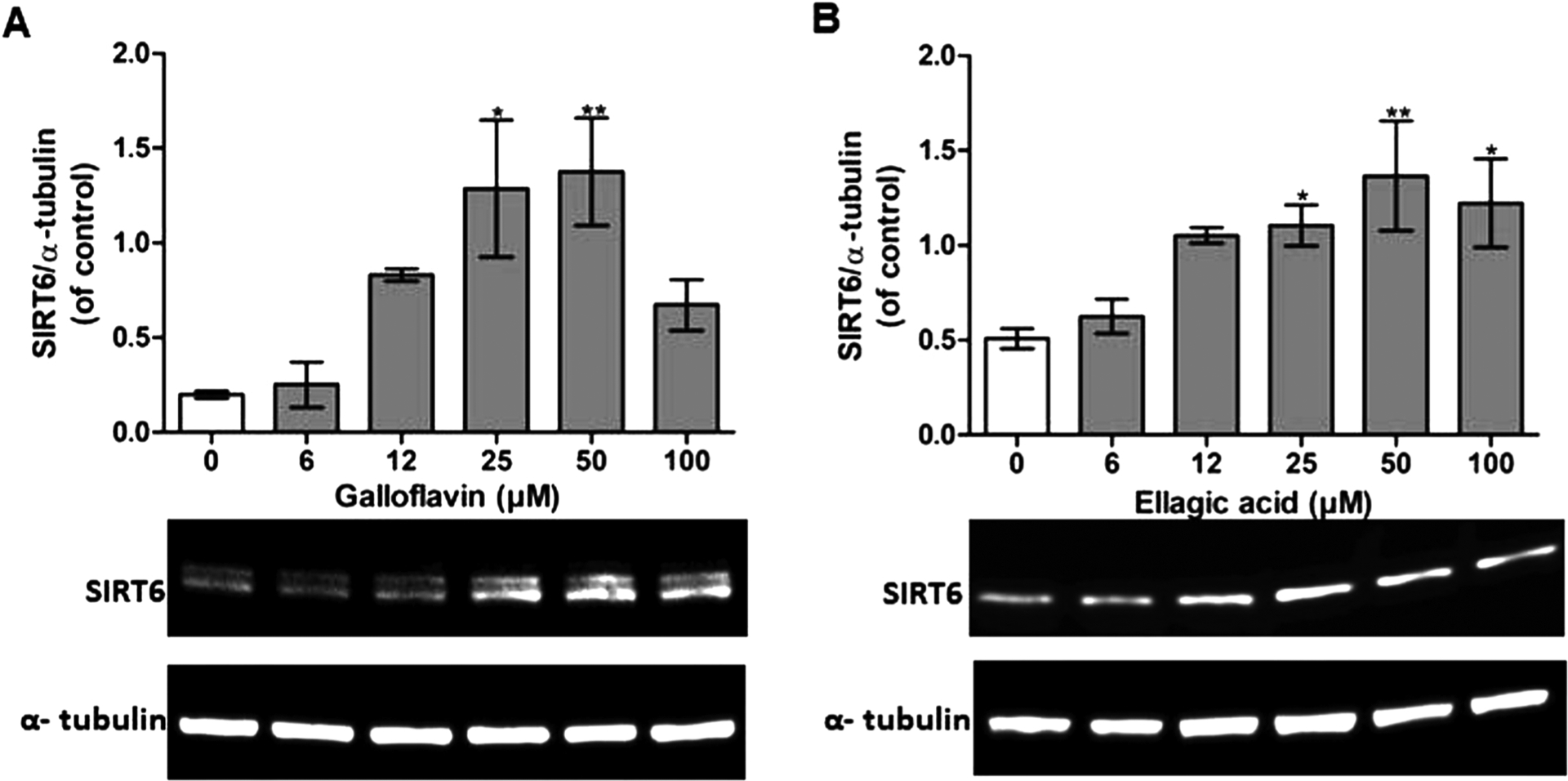
Galloflavin or Ellagic acid up-regulated SIRT6 expression in Caco2 cells. (A, B) Cells were exposed to 0.5 % DMSO control (light gray bars) or various concentrations of Galloflavin or Ellagic acid (gray bars) for 24-h. The effect of treatment on the SIRT6 expression was determined with one way-ANOVA with Bonferroni and Dunnett post hoc test by comparing treated groups to DMSO control groups. Values are expressed as mean ± SEM of three independent experiments (*p values < 0.05 vs. control, **p values < 0.01 vs. control).

Both Galloflavin and Ellagic acid showed a statistically significant increase in SIRT6 expression after 24-h exposure (Figs. 6 and S15). Galloflavin increased SIRT6 expression statistically significantly with 3.5-fold up-regulation at concentrations of 25–50 μM (Fig. 6A). Treatment with Ellagic acid resulted in a statistically significant increase with 2.5-fold maximal up-regulation at various concentrations ranging from 25 μM to 100 μM (Fig. 6B).

### 3.4. The role of Galloflavin and Ellagic acid in glucose metabolism in Caco2 cells

**Fig. 7.**
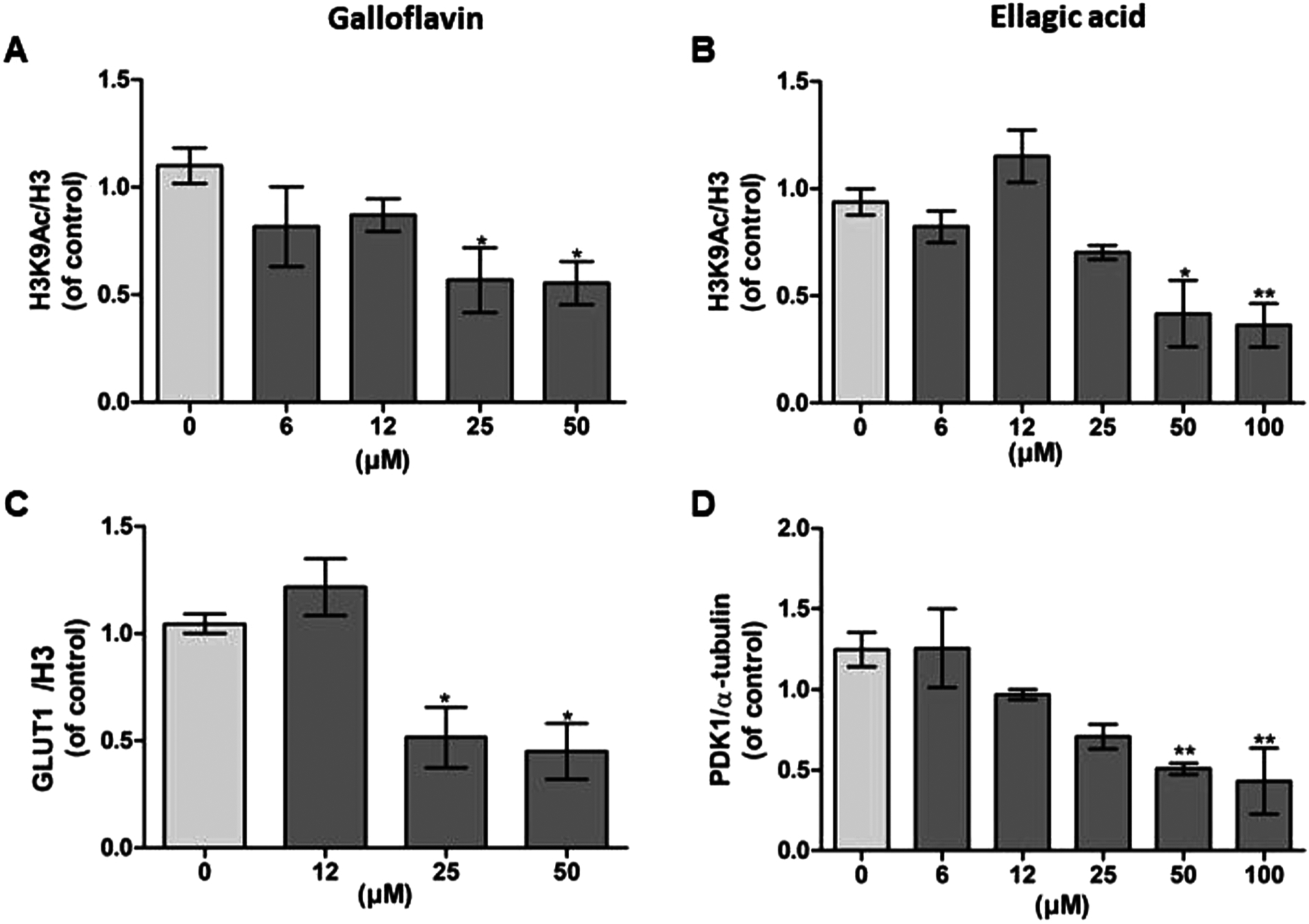
Galloflavin and Ellagic acid affected acetylation levels of H3K9Ac and the expression of SIRT6 target genes GLUT1 and PDK1. (A, B) H3K9Ac levels after Galloflavin and Ellagic acid treatments. (C) GLUT1 expression after Galloflavin treatment. (D) PDK1 expression after Ellagic acid treatment. Caco2 cells were treated with DMSO control (light gray bars) or different concentration of compounds (gray bars) for 24-h. Results are shown as a fold change compared to the control value where ratio between the protein and corresponding loading control is calculated. The effect of treatment on H3K9Ac levels or GLUT1/PDK1 expression was determined with one way-ANOVA with Bonferroni and Dunnett post hoc test by comparing treated groups to DMSO control groups. Data represents the mean ± SEM of three independent experiments (*p < 0.05 vs. control, **p < 0.01 vs. control and ***p < 0.001 vs. control).

In order to investigate the possible role of Galloflavin and Ellagic acid on HIF1α target genes, we analyzed the expression of GLUT1 and PDK1 in addition to glucose uptake and lactate production. Since SIRT6 functions as H3K9 deacetylase to silence GLUT1 and PDK1 expression [8], we also determined the protein levels of H3K9Ac in cells. Both Galloflavin and Ellagic acid decreased the levels statistically significantly with the higher concentrations compared to control (Figs. 7A and B, S16). The expression of GLUT1 protein decreased after Galloflavin treatment, and PDK1 declined after Ellagic acid treatment in Caco2 cells (Figs. 7C and D, S16).

**Fig. 8.**
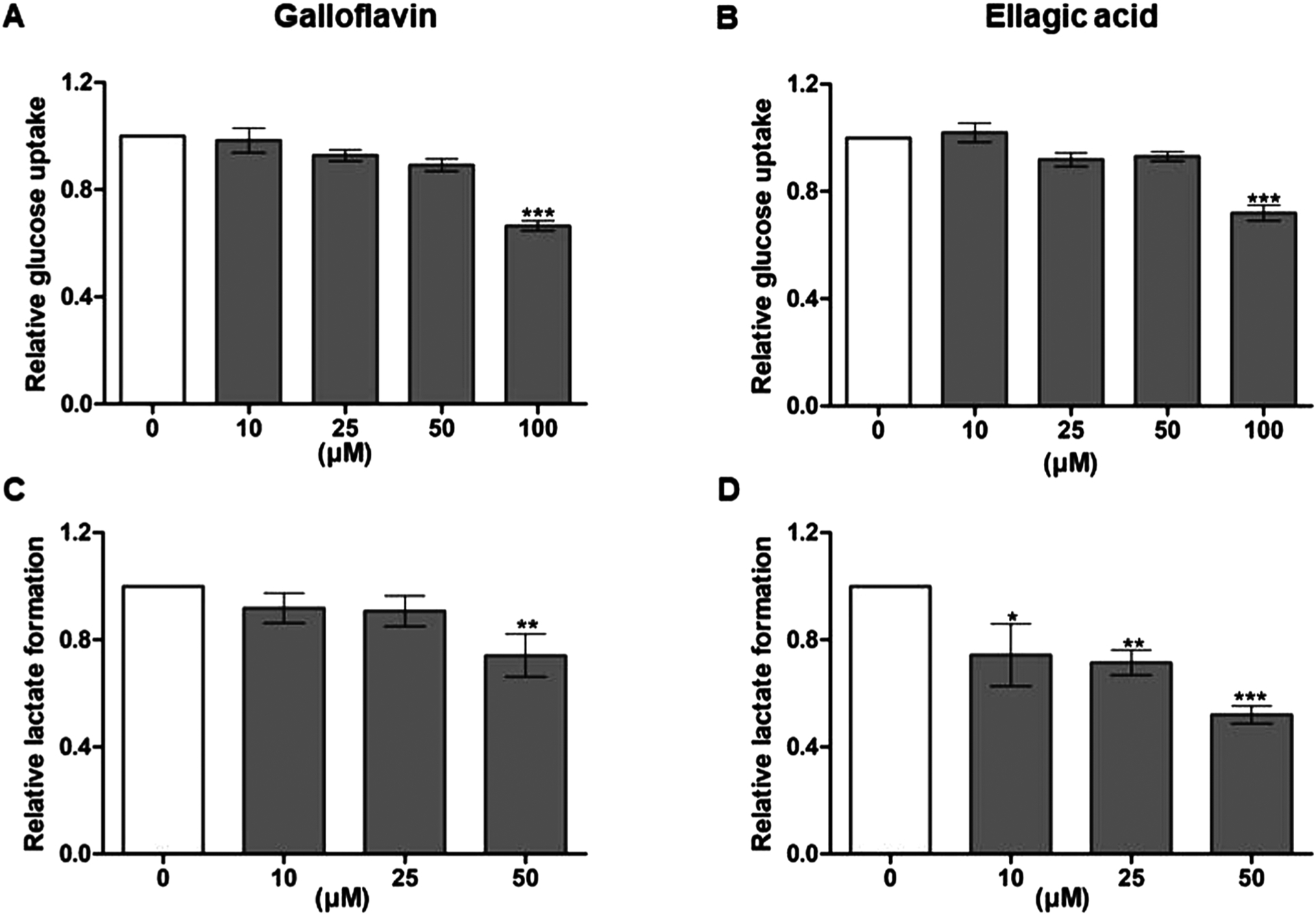
Galloflavin and Ellagic acid decreased (A, B) glucose uptake and (C, D) lactate production. Caco2 cells were exposed to DMSO control (light gray bars) or various concentrations of compounds (gray bars) for 24-h. Data represent the mean ± SEM of three independent experiments, and the statistical analysis was carried out with one way-ANOVA with Bonferroni and Dunnett post hoc test by comparing treated groups to DMSO control groups (*p < 0.05 vs. control, **p < 0.01 vs. control and ***p < 0.001 vs. control).

Both compounds appeared to reduce statistically significantly glucose uptake at 100 μM concentration (Fig. 8A and B). Interestingly, Galloflavin is a known inhibitor of LDH [27], and showed significant effects on lactate production at 50 μM concentration (Fig. 8C), whereas Ellagic acid inhibited the reaction in a dose-dependent manner at concentrations ranging from 10 μM to 50 μM (Fig. 8D).

## 4. Discussion

Some natural compounds such as long-chain fatty acids [28], and synthetic compounds have been identified as SIRT6 activators [29,30]. Moreover, previous studies demonstrated that biochemicals also found in berries can modulate SIRT6 activity, for example, anthocyanidins, including Cyanidin, induced SIRT6 activation [10,31]. In this study we showed that tannins, the most abundant biochemicals found in berries, stimulate SIRT6 activity. Galloflavin and Ellagic acid increased SIRT6 activity even more potently than anthocyanidins, but interestingly they have the opposite effect on SIRT1–3.

Sirtuins have a large hydrophobic pocket where compounds are shown to bind based on the snapshots of x-ray structures [6,18–20, 29–31]. This pocket was applied in molecular docking studies to examine interactions of compounds with SIRT6 and SIRT1–3. Our results indicated that Galloflavin and Ellagic acid were docked in the region of the pocket where they were able to form favorable interactions with SIRT6.

Many studies on Galloflavin and Ellagic acid demonstrated their anti-cancer properties [32,33]. For instance, both compounds inhibited cancer cell growth in addition to many metastatic processes such as tumor cell migration and angiogenesis [32,34]. Galloflavin and Ellagic acid have also shown anti-cancer potential in colon cancer cell lines [35, 36], therefore we used Caco2 cell line to evaluate their effects on the proliferation and expression levels of SIRT6 and SIRT6 associated proteins involved in cancer development.

In the study Galloflavin and Ellagic acid did not increase cell death in the cells, however, compounds induced a decrease in relative cell numbers indicating the effect on proliferation. In the cell cycle analyses, we observed only a minor increase in the percentage of the Caco2 cells in S phase after the Ellagic acid treatment but there were no other changes detected. Thus Ellagic acid may affect cell division without arresting the cell cycle. Our results are in line with the results by González-Sarrías et al. [35] who showed that Ellagic acid has only minor effect on the proportion of cells in different cell cycle phases. Interestingly, studies on other cell lines indicated that Ellagic acid inhibited the proliferation that is mainly mediated by arresting the cell cycle in the G_0_/G_1_ phase [37, 38].

We observed that both compounds induced an increase in SIRT6 expression on cells. A downregulation of SIRT6 is associated with many cancers [2,7] indicating a role as a tumor suppressor [1]. However, the role depends on the type of cancer since SIRT6 can also promote tumorigenesis [39]. SIRT6 gene is deleted in 29 % of colorectal cancer cell lines [40], however, SIRT6 overexpression has also shown to correlate with poor prognosis and worse overall survivals [41]. The reason for this dual role is not well understood, one reason might be the complexity of the SIRT6 target proteins but also both diversity and the high mutation frequency of different colorectal cancers [42]. In order to see a big picture, it would be important to screen compounds effect on SIRT6 in a panel of several colon cancer cell lines.

**Fig. 9.**
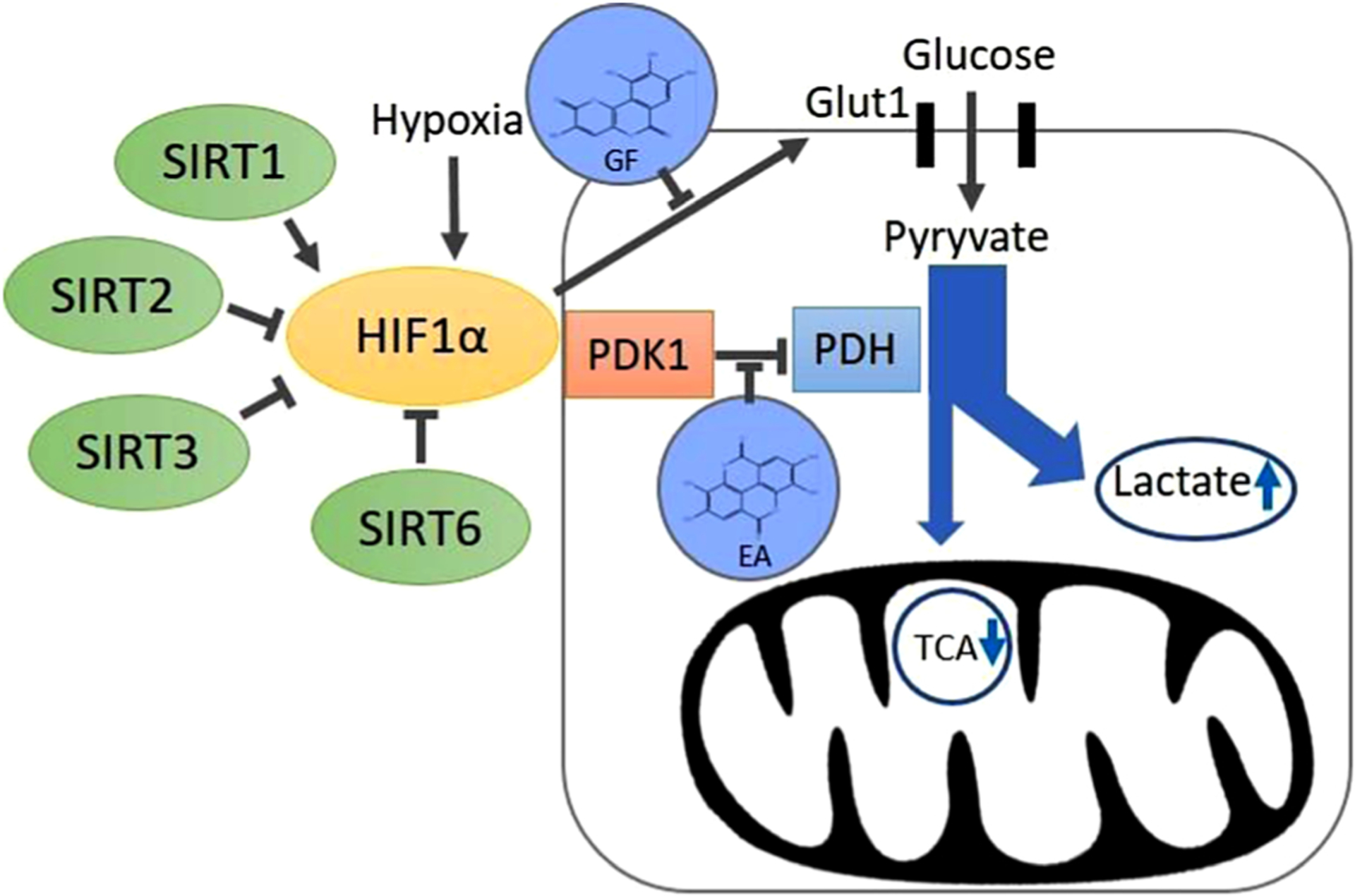
Schematic presentation metabolic reprogramming results in abnormal glycolysis in cancer cell. Process involves the uptake of high levels of glucose, enhanced glycolysis, and the metabolism of pyruvate to lactic acid rather than enter to tricarboxylic acid (TCA). Sirtuins regulated the expression of key genes, pyruvate dehydrogenase kinase 1 (PDK1) and glucose transporter 1 (GLUT1) involved in the process through hypoxia-inducible factor 1α (HIF1α). Galloflavin (GF) decreased the expression of GLUT1 and reduced glucose uptake while Ellagic acid (EA) downregulated PDK1 which subsequently decreased lactate production through pyruvate dehydrogenase (PDH).

In this study Galloflavin and Ellagic acid decreased the expression levels of SIRT6 target proteins GLUT1 and PDK1 which are associated with H3K9 deacetylation activity. These enzymes are overexpressed in various cancerous tissues and are linked to cancer-associated metabolic dysregulation [43,44]. Among the GLUT family, GLUT1 provides glucose to satisfy the extra energy requirements of cancer cells. PDK1 inactivates pyruvate dehydrogenase (PDH) and diverts pyruvate from the mitochondrial tricarboxylic acid (TCA) cycle to glycolysis with subsequent increase in lactate production (Fig. 9). This metabolic shift from TCA to glycolysis, which is a hallmark of cancer, is regulated by HIF1α. SIRTs have a pivotal role in HIF1α regulation while the regulatory role also depends on the SIRT subtype. SIRT1 stabilized HIF1α protein via many mechanisms, whereas SIRT2, SIRT3 and SIRT6 caused a decrease in HIF1α activity [45,46].

Galloflavin and Ellagic acid inhibited the function of SIRT1–3. Their inhibition indicates elevated HIF1α activation and glycolysis [45]. Even so, Galloflavin and Ellagic acid showed reduced glucose uptake and lactate production, which may be mediated via SIRT6 activation that functions as HIF1α repressor reducing the expression of many glycolysis-related proteins such as LDH, GLUT1, phosphoglycerate kinase (PGK1), glucose-6-phosphate isomerase (GPI), and phosphofructokinase 1 (PFK-1) [7,8]. Both compounds can act also through multiple other signaling pathways indicating multi-faced effects [34–36,38]. Overall, previous studies indicated that there is a multi-faceted interplay between mammalian sirtuins, and an elevated rate of glycolysis occurring in cancer cells [46].

Galloflavin and Ellagic acid are the most common polyphenols in berries growing in Artic regions. Bioactive compounds in berries appear to act through cell signaling pathways. The results of this study demonstrated that Galloflavin and Ellagic acid affected SIRT6 activity and the expression of proteins that are associated with cancer development. The compounds have also effects in glucose metabolism. Taken together, compounds targeting SIRT6 activity may provide a new approach in the development of anti-cancer therapy.

## Supplementary Material

Supplemental

## References

[1] Kanfi Y, Naiman S, Amir G, Peshti V, Zinman G, Nahum L, , The sirtuin SIRT6 regulates lifespan in male mice, Nature 483 (2012) 218–221, 10.1038/nature10815. 22367546

[2] Etchegaray JP, Zhong L, Mostoslavsky R, The histone deacetylase SIRT6: at the crossroads between epigenetics, metabolism and disease, Curr. Top. Med. Chem 13 (2013) 2991–3000, 10.2174/15680266113136660213. 24171769

[3] Michishita E, McCord RA, Berber E, Kioi M, Padilla-Nash H, Damian M, , SIRT6 is a histone H3 lysine 9 deacetylase that modulates telomeric chromatin, Nature 452 (2008) 492–496, 10.1038/nature06736. 18337721 PMC2646112

[4] Michishita E, McCord RA, Boxer LD, Barber MF, Hong T, Gozani O, , Cell cycle-dependent deacetylation of telomeric histone H3 lysine K56 by human SIRT6, Cell Cycle 8 (2009) 2664–2666. 19625767 10.4161/cc.8.16.9367 PMC4474138

[5] Mao Z, Hine C, Tian X, Van Meter M, Au M, Vaidya A, , SIRT6 promotes DNA repair under stress by activating PARP1, Science 332 (2011) 1443–1446, 10.1126/science.1202723. 21680843 PMC5472447

[6] Jiang H, Khan S, Wang Y, Charron G, He B, Sebastian C, , SIRT6 regulates TNF-α secretion through hydrolysis of long-chain fatty acyl lysine, Nature 496 (2013) 110–113, 10.1038/nature12038. 23552949 PMC3635073

[7] Lombard DB, Schwer B, Alt FW, Mostoslavsky R, SIRT6 in DNA repair, metabolism and ageing, J. Intern. Med 263 (2008) 128–141, 10.1111/j.1365-2796. 18226091 PMC2486832

[8] Zhong L, D’Urso A, Toiber D, Sebastian C, Henry RE, Vadysirisack DD, , The histone deacetylase Sirt6 regulates glucose homeostasis via Hif1alpha, Cell 140 (2010) 280–293, 10.1016/j.cell.2009.12.041. 20141841 PMC2821045

[9] Dominy JE Jr., Lee Y, Jedrychowski MP, Chim H, Jurczak MJ, Camporez JP, , The deacetylase Sirt6 activates the acetyltransferase GCN5 and suppresses hepatic gluconeogenesis, Mol. Cell 48 (2012) 900–913, 10.1016/j.molcel.2012.09.030. 23142079 PMC3534905

[10] Rahnasto-Rilla M, Tyni J, Huovinen M, Jarho E, Kulikowicz T, Ravichandran S, , Natural polyphenols as sirtuin 6 modulators, Sci. Rep 8 (2018) 4163, 10.1038/s41598-018-22388-5. 29515203 PMC5841289

[11] Smeriglio A, Barreca D, Bellocco E, Trombetta D, Proanthocyanidins and hydrolysable tannins: occurrence, dietary intake and pharmacological effects, Br. J. Pharmacol 174 (2017) 1244–1262, 10.1111/bph.13630. 27646690 PMC5429339

[12] Ferlemi AV, Lamari FN, Berry leaves: an alternative source of bioactive natural products of nutritional and medicinal value, Antioxidants Basel (Basel) 5 (2016) 17, 10.3390/antiox5020017. PMC4931538 27258314

[13] Misikangas M, Pajari AM, Päivärinta E, Oikarinen SI, Rajakangas J, Marttinen M, , Three Nordic berries inhibit intestinal tumorigenesis in multiple intestinal neoplasia/+ mice by modulating beta-catenin signaling in the tumor and transcription in the mucosa, J. Nutr 137 (2007) 2285–2290, 10.1093/jn/137.10.2285. 17885012

[14] Rahnasto-Rilla M, Kokkola T, Jarho E, Lahtela-Kakkonen M, Moaddel R, N-acylethanolamines bind to SIRT6, Chem. Bio. Chem 17 (2016) 77–81, 10.1002/cbic.201500482. PMC4813509 26607666

[15] Rahnasto-Rilla M, Lahtela-Kakkonen M, Moaddel R, Sirtuin 6 (SIRT6) activity assays, Methods Mol. Biol 1436 (2016) 259–269, 10.1007/978-1-4939-3667-0_17. 27246220 PMC5004593

[16] Rahnasto-Rilla MK, McLoughlin P, Kulikowicz T, Doyle M, Bohr VA, Lahtela-Kakkonen M, , The identification of a SIRT6 activator from brown algae Fucus distichus, Mar. Drugs 15 (2017) 190, 10.3390/md15060190. 28635654 PMC5484140

[17] Kiviranta PH, Leppanen J, Rinne VM, Suuronen T, Kyrylenko O, Kyrylenko S, , N-(3-(4-Hydroxyphenyl)-propenoyl)-amino acid tryptamides as SIRT2 inhibitors, Bio. Org. Med. Chem. Lett 17 (2007) 2448–2451, 10.1016/j.bmcl.2007.02.023. 17329104

[18] Zhao X, Allison D, Condon B, Zhang F, Gheyi T, Zhang A, , The 2.5 Å crystal structure of the SIRT1 catalytic domain bound to nicotinamide adenine dinucleotide (NAD+) and an indole (EX527 analogue) reveals a novel mechanism of histone deacetylase inhibition, J. Med. Chem 56 (2013) 963–969, 10.1021/jm301431y. 23311358

[19] Rumpf T, Schiedel M, Karaman B, Roessler C, North BJ, Lehotzky A, , Selective Sirt2 inhibition by ligand-induced rearrangement of the active site, Nat. Commun 6 (2015) 6263, 10.1038/ncomms7263. 25672491 PMC4339887

[20] Gertz M, Fischer F, Nguyen GTT, Lakshminarasimhan M, Schutkowski M, Weyand M, Steegborn C, Ex-527 inhibits Sirtuins by exploiting their unique NAD +-dependent deacetylation mechanism, Proc. Natl. Acad. Sci. U. S. A 110 (2013) 2772–2781, 10.1073/pnas.1303628110. PMC3725051 23840057

[21] Roos K, Wu C, Damm W, Reboul M, Stevenson JM, Lu C, , OPLS3e: extending force field coverage for drug-like small molecules, J. Chem. Theory Comput 15 (2019) 1863–1874, 10.1021/acs.jctc.8b01026. 30768902

[22] Huovinen M, Loikkanen J, Myllynen P, Vähäkangas KH, Characterization of human breast cancer cell lines for the studies on p53 in chemical carcinogenesis, Toxicol. In Vitro 25 (2011) 1007–1017, 10.1016/j.tiv.2011.03.018. 21457773

[23] Yamamoto N, Ueda-Wakagi M, Sato T, Kawasaki K, Sawada K, Kawabata K, , Measurement of glucose uptake in cultured cells, Curr. Protoc. Pharmacol 71 (2015), 10.1002/0471141755.ph1214s71. 26646194

[24] Losso JN, Bansode RR, Trappey A 2nd, Bawadi HA, Truax R, In vitro anti-proliferative activities of ellagic acid, J. Nutr. Biochem 15 (2004) 672–678. 15590271 10.1016/j.jnutbio.2004.06.004

[25] Han X, Sheng X, Jones HM, Jackson AL, Kilgore J, Stine JE, , Evaluation of the anti-tumor effects of lactate dehydrogenase inhibitor galloflavin in endometrial cancer cells, J. Hematol. Oncol 8 (2015) 2, 10.1186/s13045-014-0097-x. 25631326 PMC4316809

[26] Moniot S, Weyand M, Steegborn C, Structures, substrates, and regulators of Mammalian sirtuins - opportunities and challenges for drug development, Front. Pharmacol 3 (2012) 16, 10.3389/fphar.2012.00016. 22363286 PMC3275776

[27] Farabegoli F, Vettraino M, Manerba M, Fiume L, Roberti M, Di Stefano G, Galloflavin, a new lactate dehydrogenase inhibitor, induces the death of human breast cancer cells with different glycolytic attitude by affecting distinct signaling pathways, Eur. J. Pharm. Sci 47 (2012) 729–738, 10.1016/j.ejps.2012.08.012. 22954722

[28] Feldman JL, Baeza J, Denu JM, Activation of the protein deacetylase SIRT6 by long-chain fatty acids and widespread deacylation by mammalian sirtuins, J. Biol. Chem 288 (2013) 31350–31356, 10.1074/jbc.C113.511261. 24052263 PMC3829447

[29] You W, Rotili D, Li TM, Kambach C, Meleshin M, Schutkowski M, Structural basis of sirtuin 6 activation by synthetic small molecules, Angew. Chem. Int. Ed. Engl 56 (2017) 1007–1011, 10.1002/anie.201610082. 27990725

[30] Huang Z, Zhao J, Deng W, Chen Y, Shang J, Song K, Identification of a cellularly active SIRT6 allosteric activator, Nat. Chem. Biol 14 (2018) 1118–1126, 10.1038/s41589-018-0150-0. 30374165

[31] You W, Zheng W, Weiss S, Chua KF, Steegborn C, Structural basis for the activation and inhibition of Sirtuin 6 by quercetin and its derivatives, Sci. Rep 9 (2019) 19176, 10.1038/s41598-019-55654-1. 31844103 PMC6914789

[32] Han X, Sheng X, Jones HM, Jackson AL, Kilgore J, Stine JE, , Evaluation of the anti-tumor effects of lactate dehydrogenase inhibitor galloflavin in endometrial cancer cells, J. Hematol. Oncol 8 (2015) 2, 10.1186/s13045-014-0097-x. 25631326 PMC4316809

[33] Ismail T, Calcabrini C, Diaz AR, Fimognari C, Turrini E, Catanzaro E, , Ellagitannins in cancer chemoprevention and therapy, Toxins 8 (2016) 151, 10.3390/toxins8050151. 27187472 PMC4885066

[34] Zhang HM, Zhao L, Li H, Xu H, Chen WW, Tao L, Research progress on the anticarcinogenic actions and mechanisms of ellagic acid, Cancer Biol. Med 11 (2014) 92–100, 10.7497/j.issn.2095-3941.2014.02.004. 25009751 PMC4069806

[35] González-Sarrías A, Espín JC, Tomás-Barberán FA, García-Conesa MT, Gene expression, cell cycle arrest and MAPK signalling regulation in Caco-2 cells exposed to ellagic acid and its metabolites, urolithins, Mol. Nutr. Food Res 53 (2009) 686–698, 10.1002/mnfr.200800150. 19437480

[36] Manerba M, Di Ianni L, Govoni M, Roberti M, Recanatini M, Di Stefano G, Lactate dehydrogenase inhibitors can reverse inflammation induced changes in colon cancer cells, Eur. Pharm. Sci 96 (2017) 37–44, 10.1016/j.ejps.2016.09.014. 27622920

[37] Chen HS, Bai MH, Zhang T, Li GD, Liu M, Ellagic acid induces cell cycle arrest and apoptosis through TGF-β/Smad3 signaling pathway in human breast cancer MCF-7 cells, Int. J. Oncol 46 (2015) 1730–1738, 10.3892/ijo.2015.2870. 25647396

[38] Li TM, Chen GW, Su CC, Lin JG, Yeh CC, Cheng KC, Chung JG, Ellagic acid induced p53/p21 expression, G1 arrest and apoptosis in human bladder cancer T24 cells, Anticancer Res. 25 (2005) 971–979. 15868936

[39] Desantis V, Lamanuzzi A, Vacca A, The role of SIRT6 in tumors, Haematologica 103 (2018) 1–4, 10.3324/haematol.2017.182675. 29290628 PMC5777184

[40] Sebastián C, Zwaans BM, Silberman DM, Gymrek M, Goren A, Zhong L, , The histone deacetylase SIRT6 is a tumor suppressor that controls cancer metabolism, Cell. 151 (2012) 1185–1199, 10.1016/j.cell.2012.10.047. 23217706 PMC3526953

[41] Geng CH, Zhang CL, Zhang JY, Gao P, He M, Li YL, Overexpression of Sirt6 is a novel biomarker of malignant human colon carcinoma, J. Cell. Biochem 119 (2018) 3957–3967, 10.1002/jcb.26539. 29227545

[42] Mouradov D, Sloggett C, Jorissen RN, Love CG, Li S, Burgess AW, , Colorectal cancer cell lines are representative models of the main molecular subtypes of primary cancer, Cancer Res. 74 (2014) 3238–3247, 10.1158/0008-5472.CAN-14-0013. 24755471

[43] Gagliardi PA, Puliafito A, Primo L, PDK1: At the crossroad of cancer signaling pathways, Semin. Cancer Biol 48 (2018) 27–35, 10.1016/j.semcancer.2017.04.014. 28473254

[44] Wang J, Ye C, Chen C, Xiong H, Xie B, Zhou J, , Glucose transporter GLUT1 expression and clinical outcome in solid tumors: a systematic review and meta-analysis, Oncotarget 8 (2017) 16875–16886, 10.18632/oncotarget.15171. 28187435 PMC5370007

[45] Takiyama Y, Haneda M, Hypoxia in diabetic kidneys, Biomed. Res. Int 2014 (2014), 837421, 10.1155/2014/837421. 25054148 PMC4094876

[46] Zwaans BMM, Lombard DB, Interplay between sirtuins, MYC and hypoxiainducible factor in cancer-associated metabolic reprogramming, Dis. Model. Mech 7 (2014) 1023–1032, 10.1242/dmm.016287. 25085992 PMC4142723

