# Supplementary material for: Effects of galloflavin and ellagic acid on sirtuin 6 and its anti-tumorigenic activities
Source: Biomed Pharmacother. Author manuscript; Available in PMC 2025 Apr 28. (PMC12036747; doi:10.1016/j.biopha.2020.110701)
Supplement: Supplemental [file NIHMS1804147-supplement-Supplemental.pdf]

Effects of Ellagic acid on Sirtuin 6 and its anti-tumorigenic activities

Minna Rahnasto-Rilla[a,1,*], Joni Järvenpää[a,1], Marjo Huovinen[a], Anna-Mari Schroderus[b], Emmi-Leena Ihantola[b], Jenni Küblbeck[a], Mohammed Khadeer[c], Ruin Moaddel[c], Maija Lahtela-Kakkonen[a]

[a]School of Pharmacy, University of Eastern Finland, 70210, Kuopio, Finland
[b]Department of Clinical Microbiology, Institute of Clinical Medicine, University of Eastern Finland, Kuopio, Finland
[c]Biomedical Research Center, National Institute on Aging, National Institutes of Health, Baltimore, Maryland, 21224, USA

[1]Equal contribution

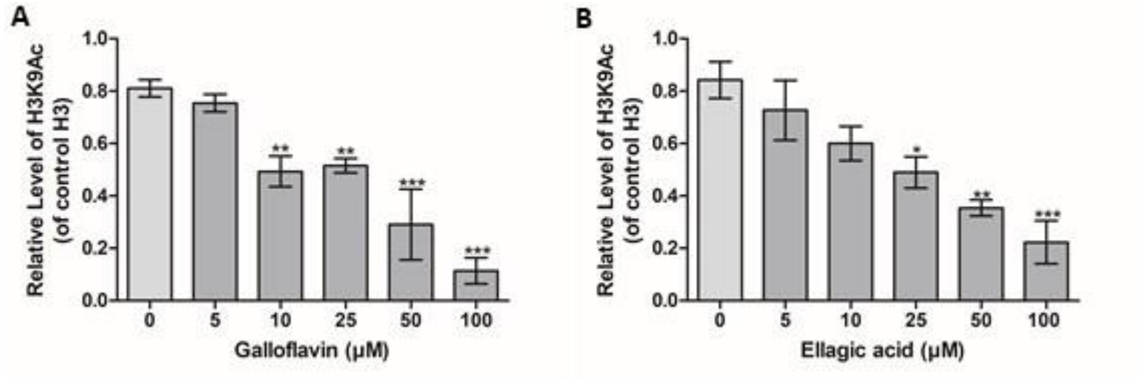

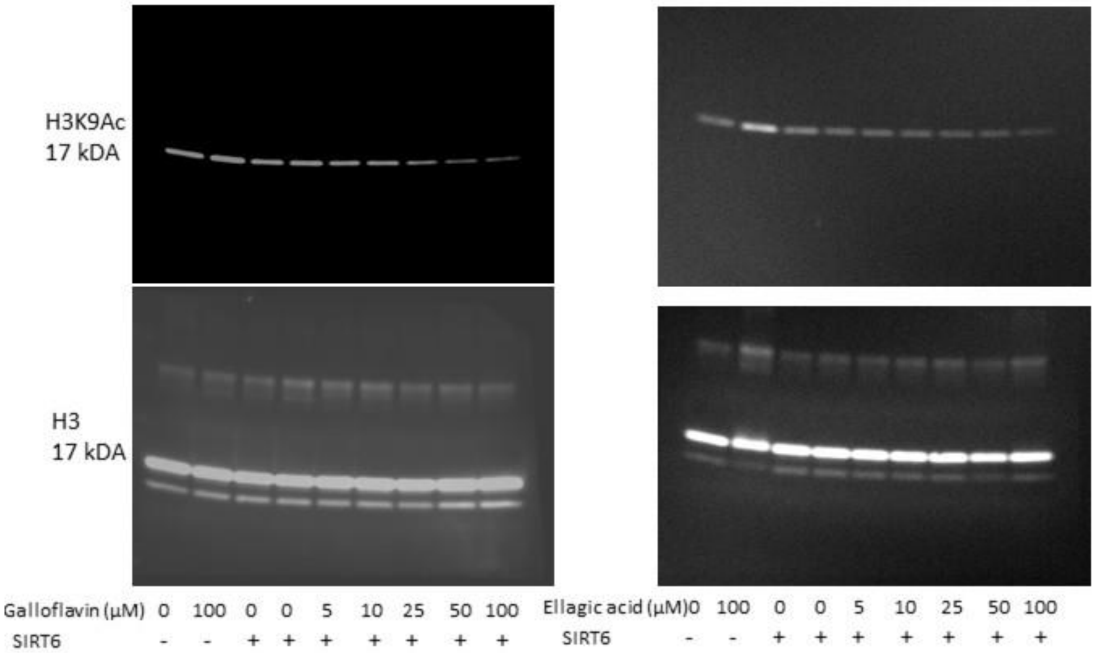

Figure S1. Relative SIRT6 H3K9Ac deacetylation activity by Immunoblotting assay and uncropped blots probed with H3K9Ac or H3. Values are expressed as mean ± standard error of mean (SEM) of three independent experiments (*p values < 0.05 vs. control, **p values < 0.01 vs. control; ***p values < 0.001 vs. control; one-way ANOVA with Bonferroni and Dunnett post hoc test).

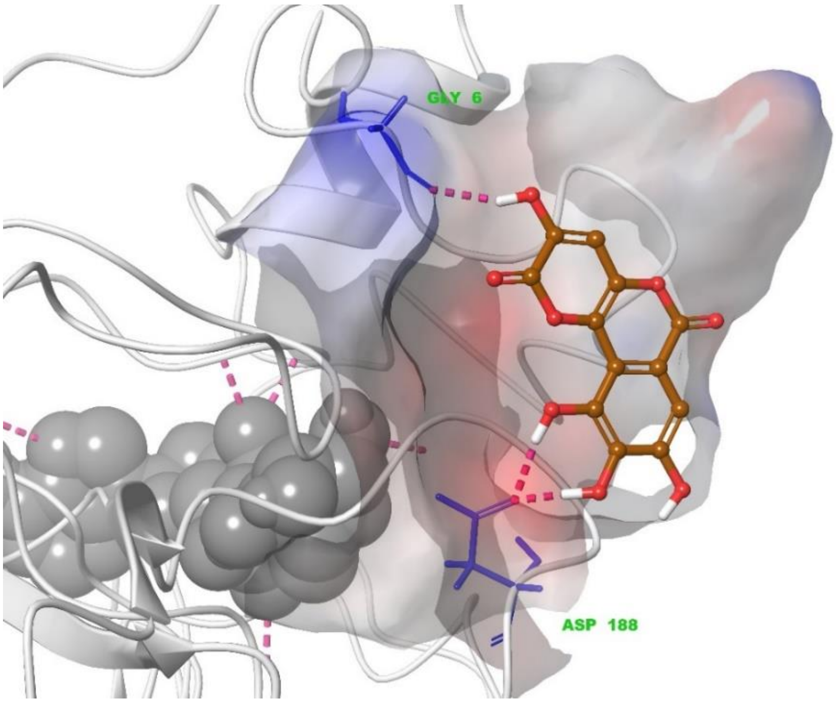

Figure S2. The docking pose of Galloflavin at SIRT6's putative activator binding site. The purple dashes indicate hydrogen bonding between Galloflavin and amino acids Gly6 and Asp188. ADP-ribose is presented with CPK model in grey. The binding site surface is colored by value of the electrostatic potential with blue showing the highest potentials and red the lowest.

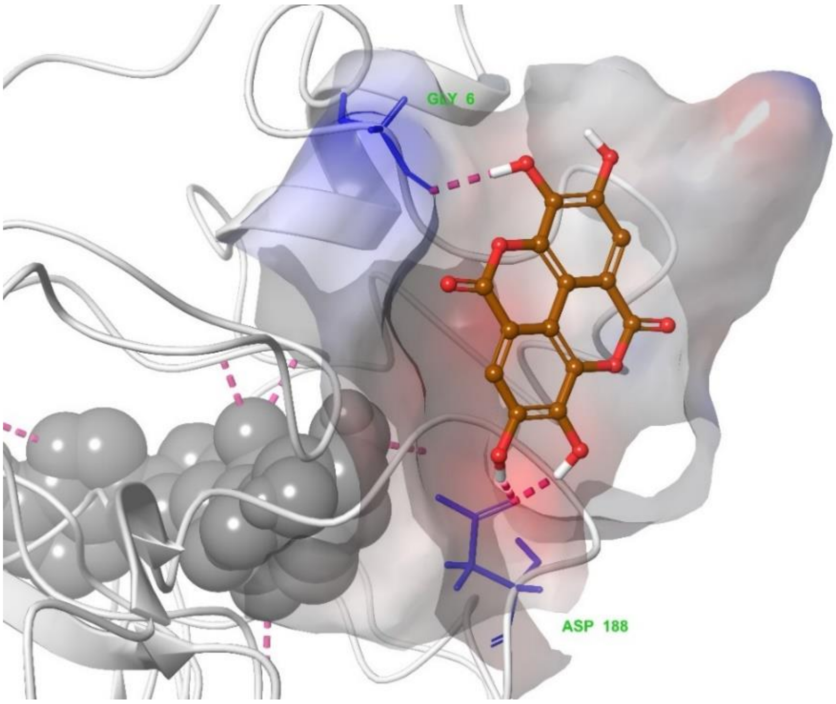

Figure S3. The docking pose of Ellagic acid at SIRT6's putative activator binding site. The purple dashes indicate hydrogen bonding between Ellagic acid and amino acids Gly6 and Asp188. ADP-ribose (Adenosine diphosphate ribose) part of cofactor NAD^{+} is presented with CPK model in grey. The binding site surface is colored by value of the electrostatic potential with blue showing the highest potentials and red the lowest.

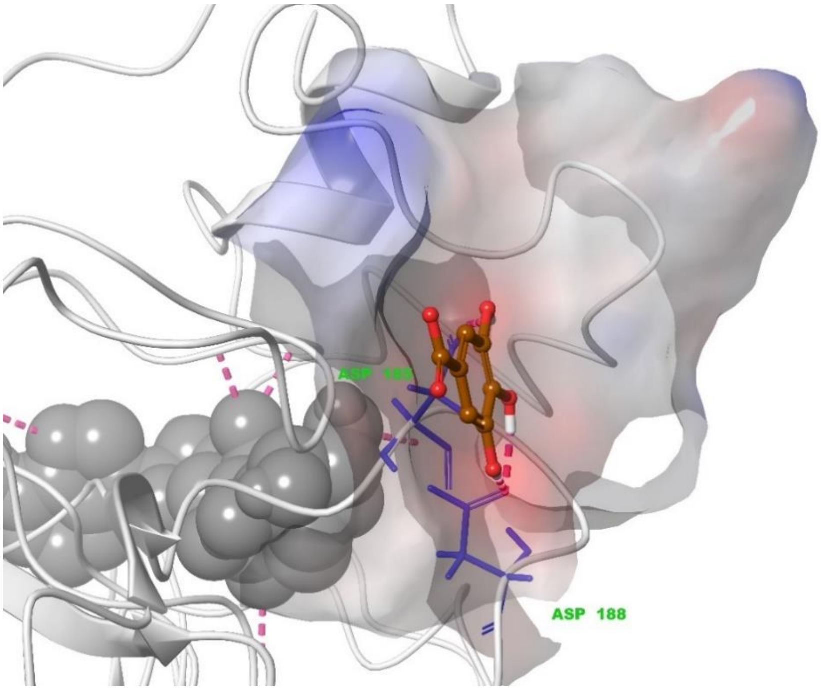

Figure S4. The docking pose of Gallic acid at SIRT6's putative activator binding site. The purple dashes indicate hydrogen bonding between Gallic acid and amino acids Asp185 and Asp188. ADP-ribose is presented with CPK model in grey. The binding site surface is colored by value of the electrostatic potential with blue showing the highest potentials and red the lowest.

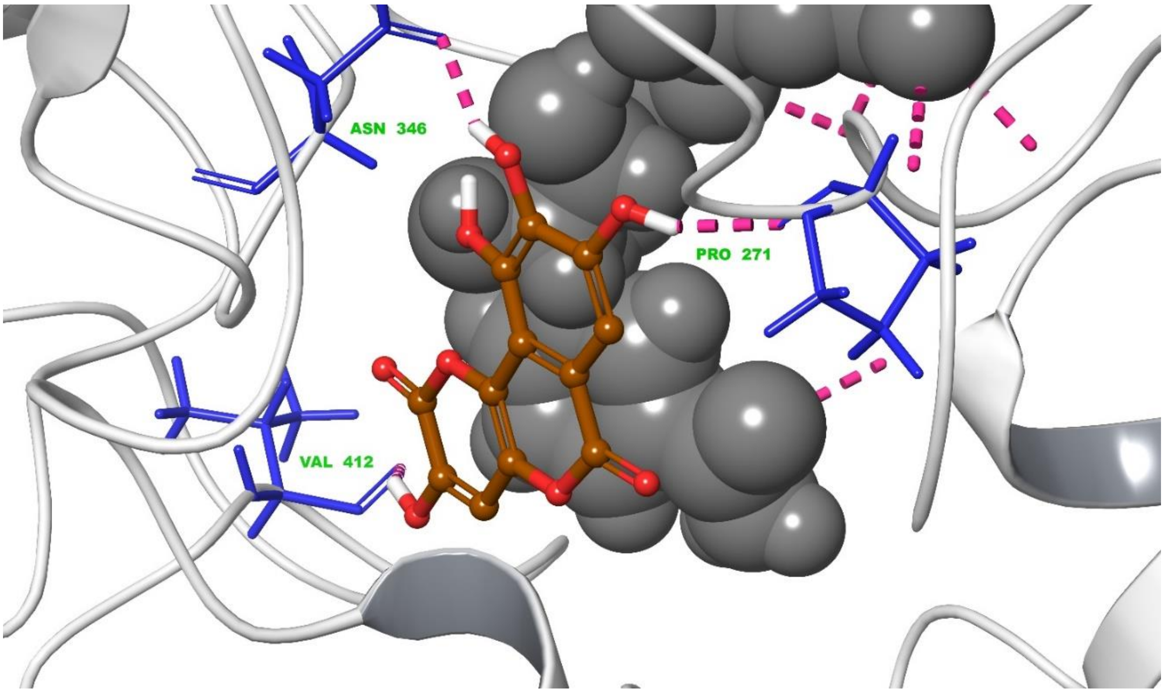

Figure S5. The docking pose of Galloflavin at SIRT1's inhibitor binding site. The purple dashes indicate hydrogen bonding between Galloflavin and amino acids Pro271, Asn346 and Val412. NAD+ is presented with CPK model in grey.

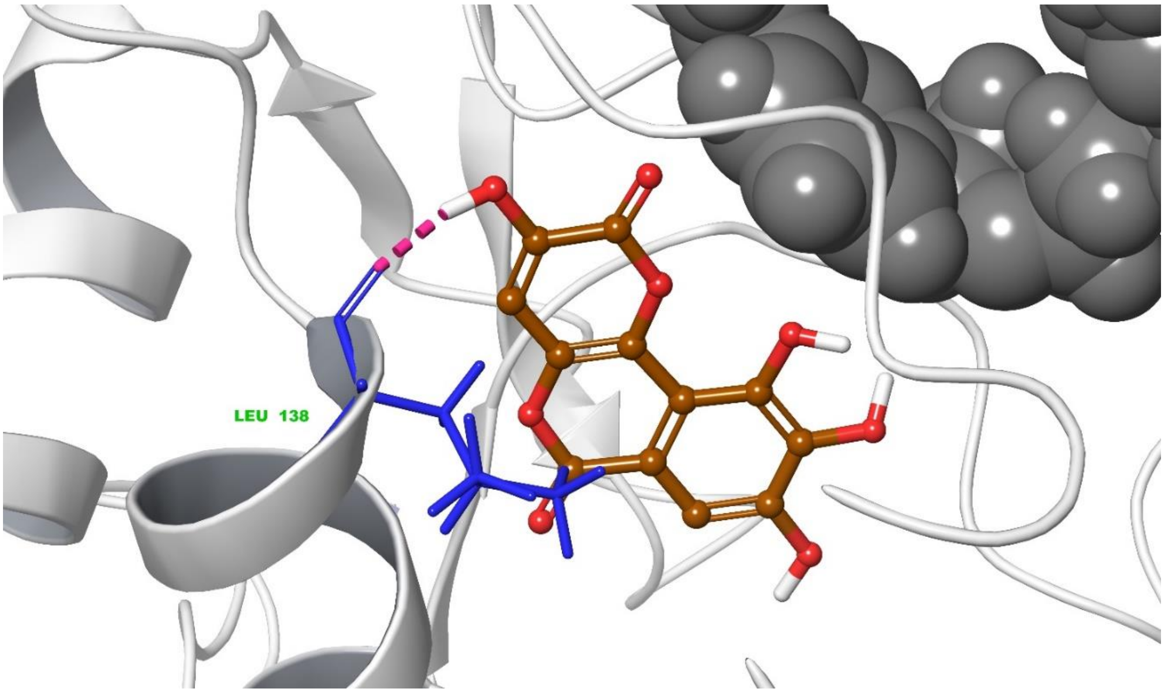

Figure S6. The docking pose of Galloflavin at SIRT2's inhibitor binding site. The purple dashes indicate hydrogen bonding between Galloflavin and amino acid Leu138. NAD^{+} is presented with CPK model in grey.

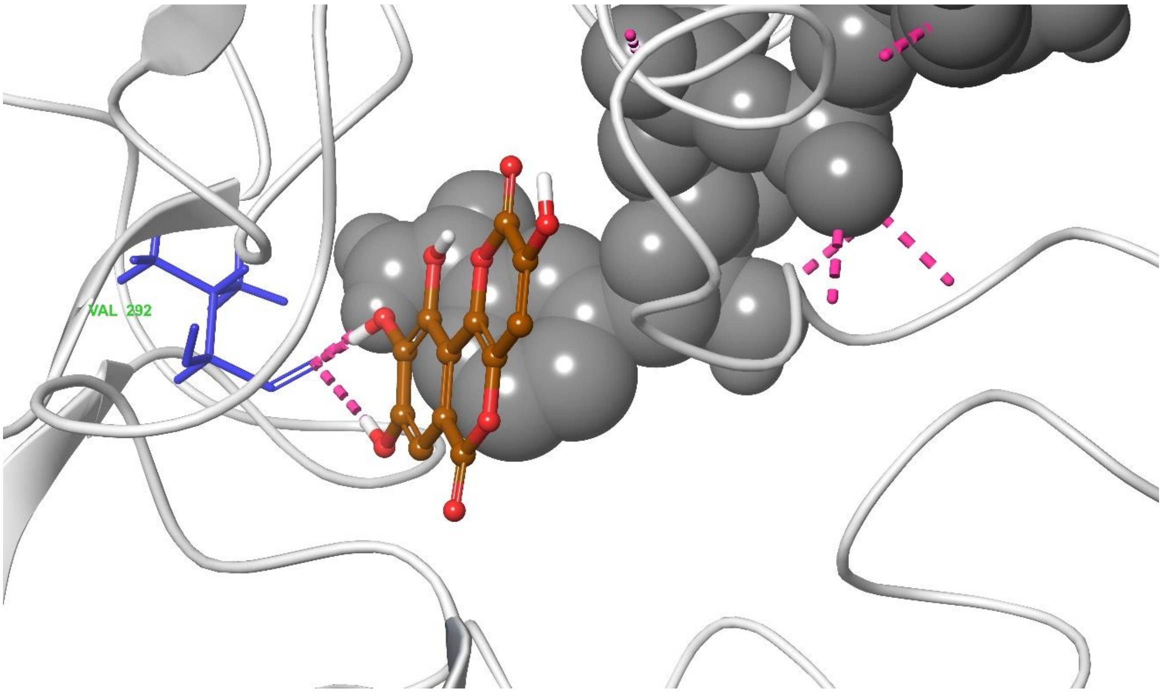

Figure S7. The docking pose of Galloflavin at SIRT3's inhibitor binding site. The purple dashes indicate hydrogen bonding between Galloflavin and amino acid Val292. NAD+ is presented with CPK model in grey.

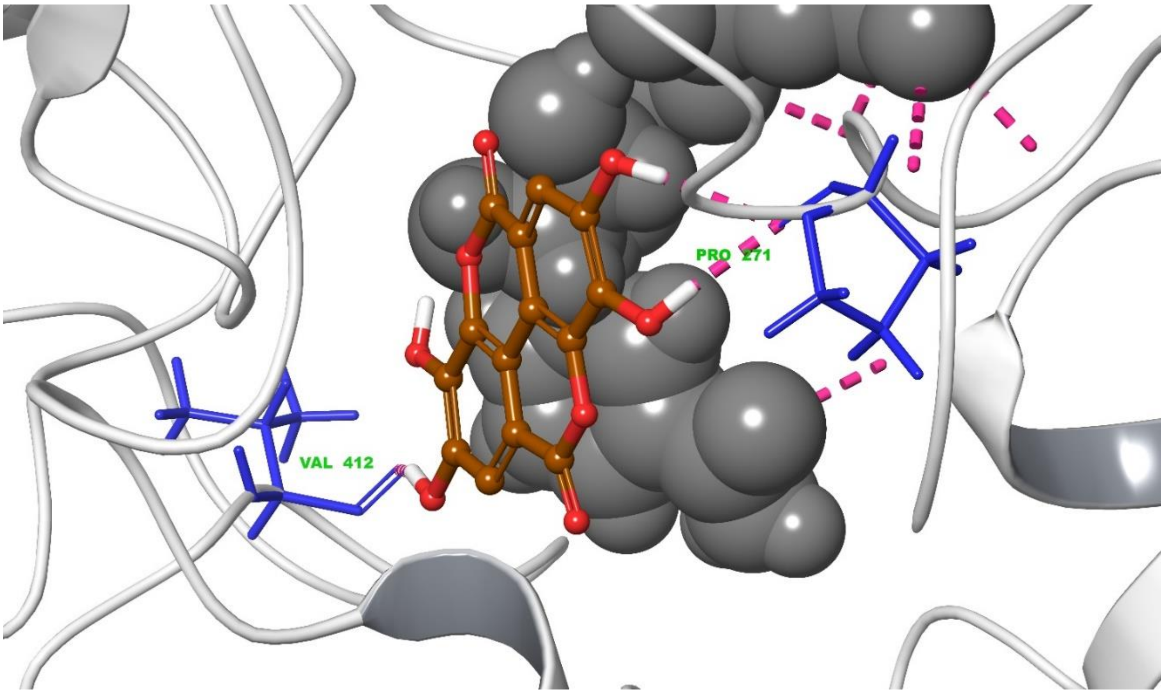

Figure S8. The docking pose of Ellagic acid at SIRT1's inhibitor binding site. The purple dashes indicate hydrogen bonding between Ellagic acid and amino acids Pro271 and Val412. NAD+ is presented with CPK model in grey.

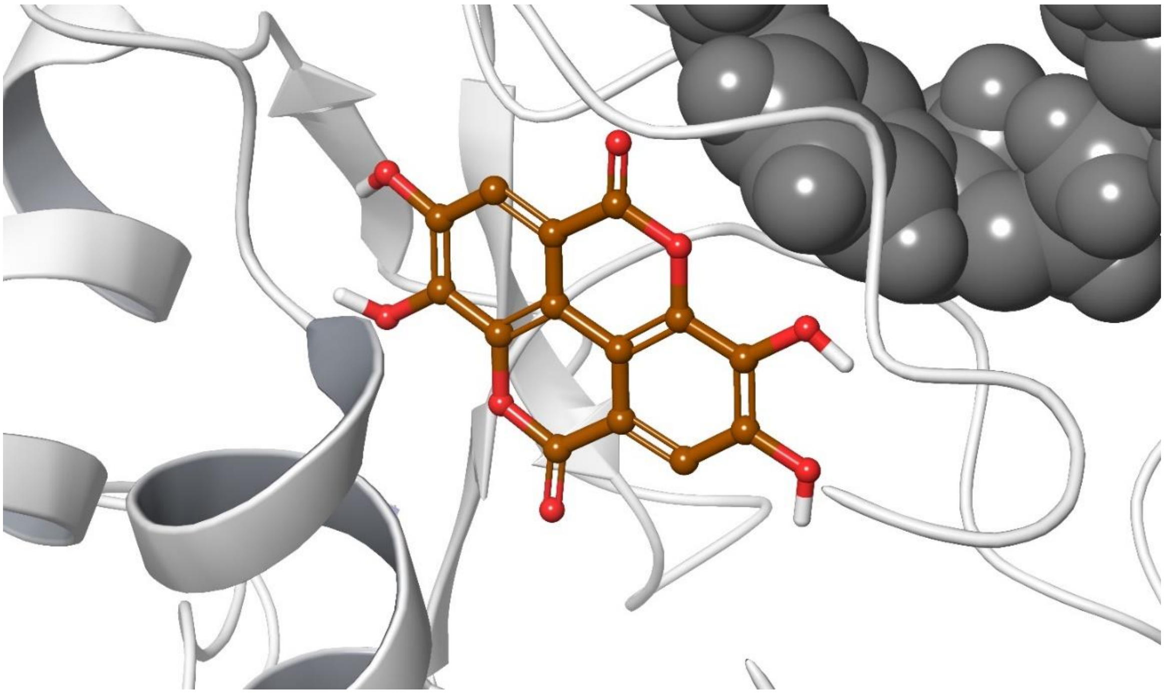

Figure S9. The docking pose of Ellagic acid at SIRT2's inhibitor binding site. NAD^+ is presented with CPK model in grey.

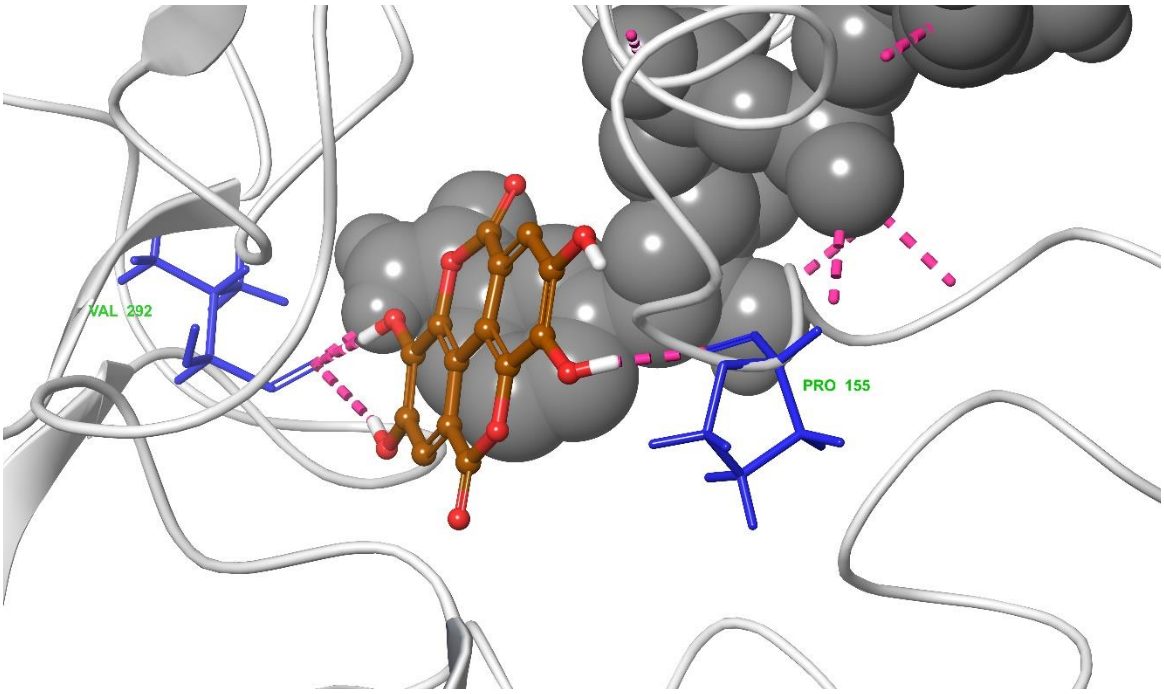

Figure S10. The docking pose of Ellagic acid at SIRT3's inhibitor binding site. The purple dashes indicate hydrogen bonding between Ellagic acid and amino acids Pro155 and Val292. NAD+ is presented with CPK model in grey.

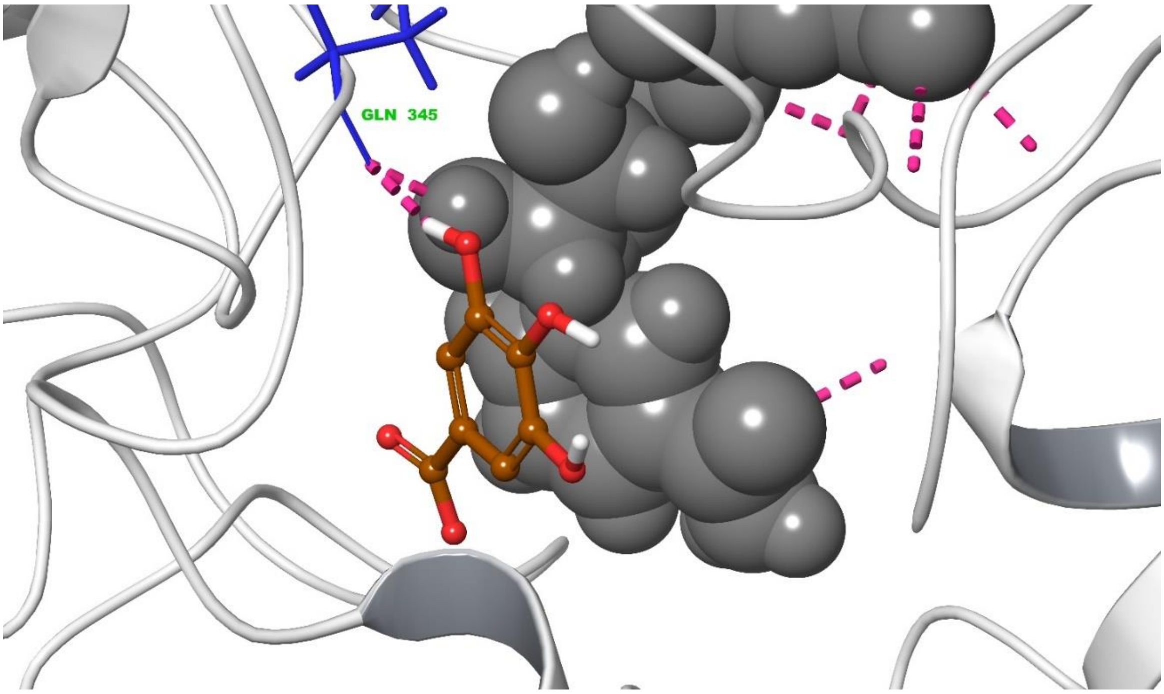

Figure S11. The docking pose of Gallic acid at SIRT1's inhibitor binding site. The purple dashes indicate hydrogen bonding between Gallic acid and amino acid Gln345. NAD+ is presented with CPK model in grey.

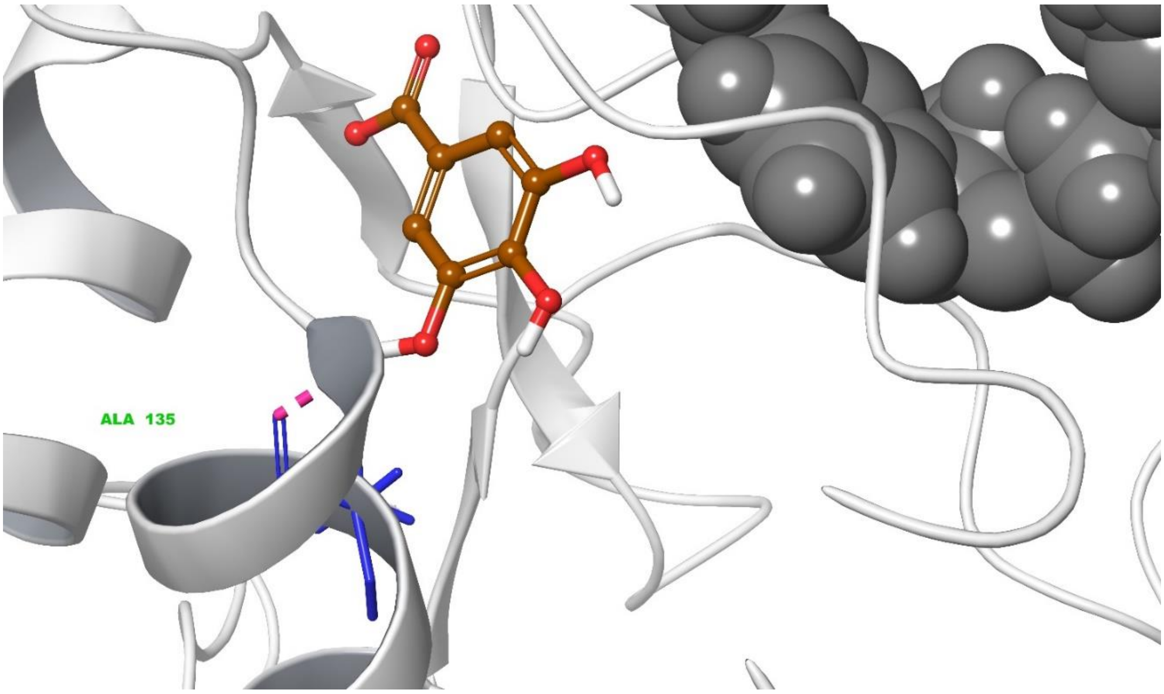

Figure S12. The docking pose of Gallic acid at SIRT2's inhibitor binding site. The purple dashes indicate hydrogen bonding between Gallic acid and amino acid Ala135. NAD+ is presented with CPK model in grey.

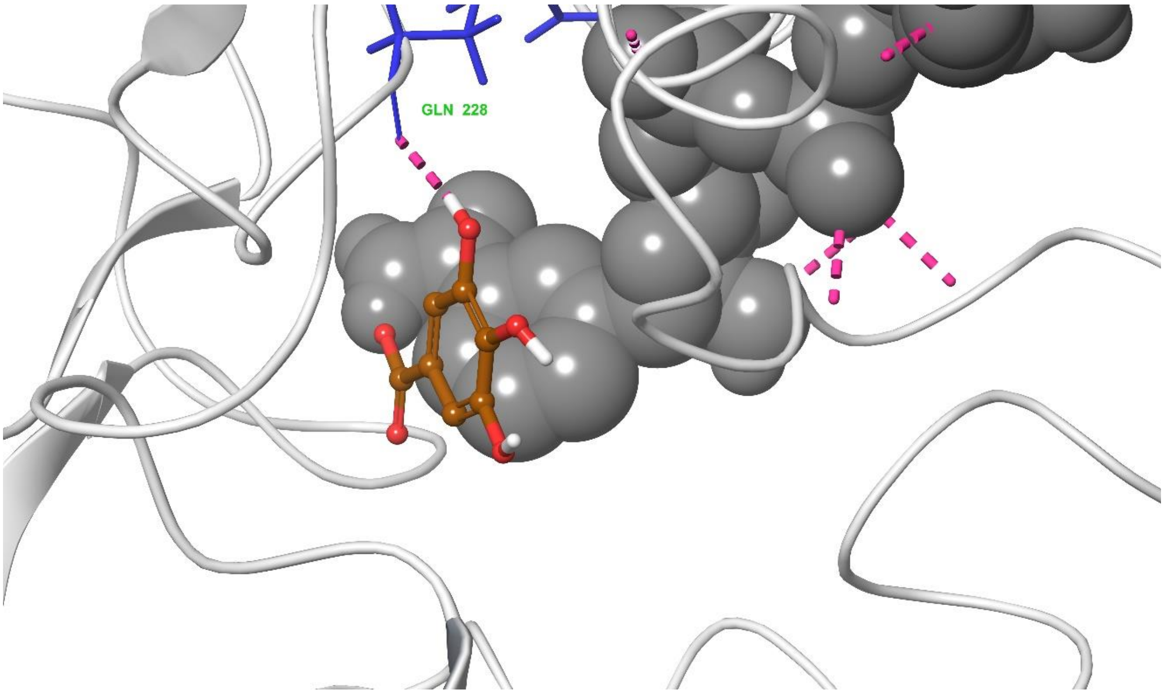

Figure S13. The docking pose of Gallic acid at SIRT3's inhibitor binding site. The purple dashes indicate hydrogen bonding between Gallic acid and amino acid Gln228. NAD+ is presented with CPK model in grey.

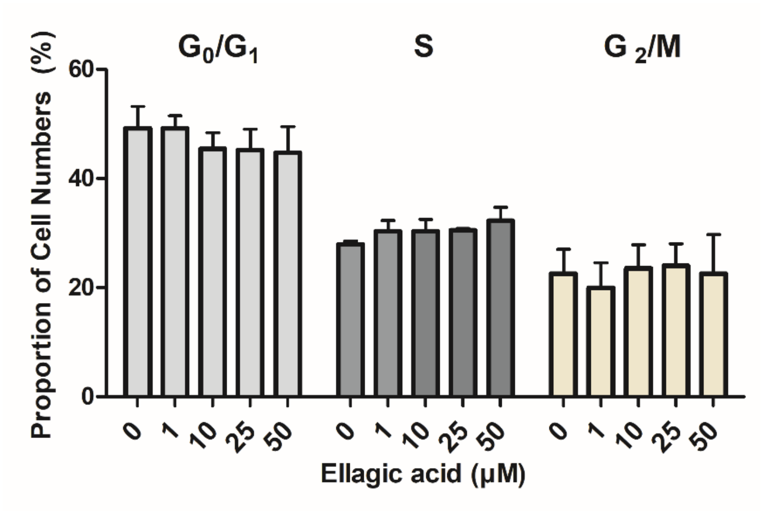

Figure S14. Treatment with Ellagic acid (1-50 µM) showed a slight decrease of cell distribution in G_0/G_1 phase and an increase of cell distribution in phase S after treatment at the highest concentrations. The percentage of cells in each cycle phase was determined by flow cytometry. Data are presented as mean values ± SEM (n=3).

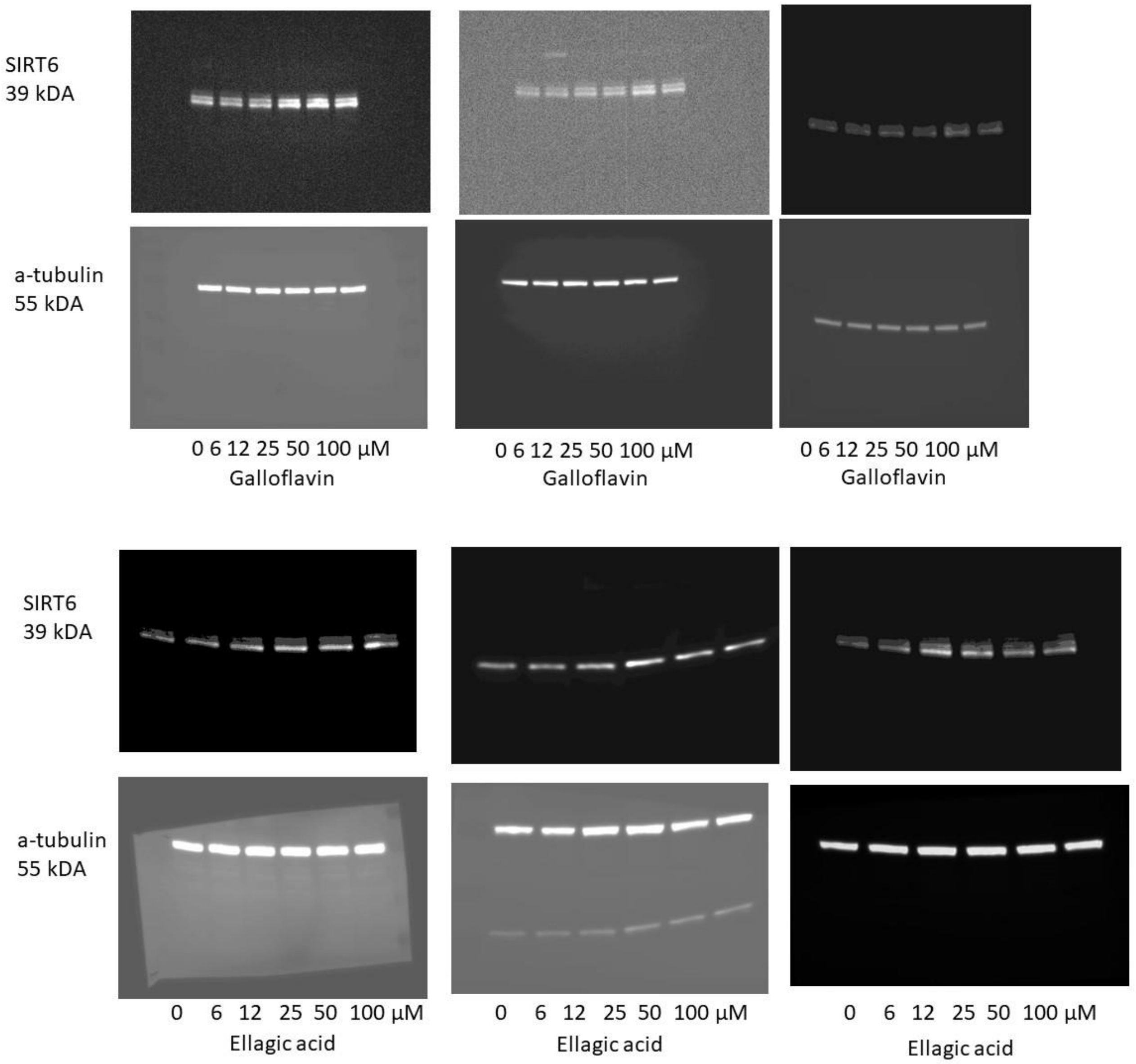

Figure S15. Uncropped blots probed with SIRT6. Densitometric analysis of protein bands was carried out using ImageJ 1.32 software and the data were normalized by α-tubulin (loading controls).

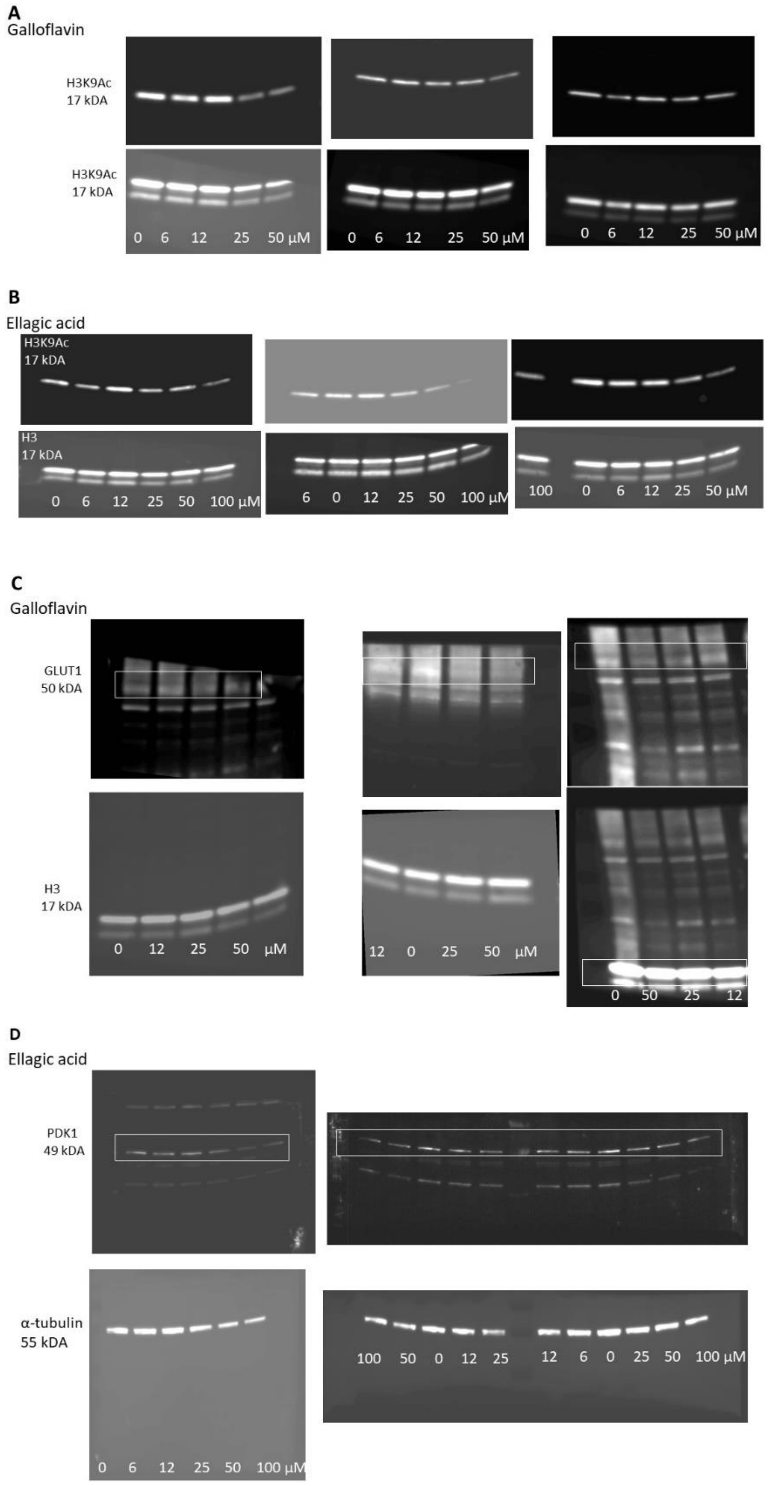

Figure S16. Uncropped blots probed with (A-B) H3K9Ac, (C) GLUT1 and (D) PDK1. Densitometric analysis of protein bands was carried out using ImageJ 1.32 software and the data were normalized by H3 or α-tubulin (loading controls).
